# Selected strategies to fight pathogenic bacteria

**DOI:** 10.1080/14756366.2022.2155816

**Published:** 2023-01-11

**Authors:** Aiva Plotniece, Arkadij Sobolev, Claudiu T. Supuran, Fabrizio Carta, Fredrik Björkling, Henrik Franzyk, Jari Yli-Kauhaluoma, Koen Augustyns, Paul Cos, Linda De Vooght, Matthias Govaerts, Juliana Aizawa, Päivi Tammela, Raivis Žalubovskis

**Affiliations:** a Latvian Institute of Organic Synthesis, Riga, Latvia; b Department of Pharmaceutical Chemistry, Faculty of Pharmacy, Riga Stradiņš University, Riga, Latvia; c Department of NEUROFARBA, Section of Pharmaceutical and Nutraceutical Sciences, University of Florence, Firenze, Italy; d Department of Drug Design and Pharmacology, Faculty of Health and Medical Sciences, Center for Peptide-Based Antibiotics, University of Copenhagen, Copenhagen East, Denmark; e Division of Pharmaceutical Chemistry and Technology, Faculty of Pharmacy, Drug Research Program, University of Helsinki, Helsinki, Finland; f Infla-Med, Centre of Excellence, University of Antwerp, Antwerp, Belgium; g Laboratory of Medicinal Chemistry, University of Antwerp, Antwerp, Belgium; h Department of Pharmaceutical Sciences, Laboratory for Microbiology, Parasitology and Hygiene (LMPH), University of Antwerp, Antwerp, Belgium; i Division of Pharmaceutical Biosciences, Faculty of Pharmacy, Drug Research Program, University of Helsinki, Helsinki, Finland; j Faculty of Materials Science and Applied Chemistry, Institute of Technology of Organic Chemistry, Riga Technical University, Riga, Latvia

**Keywords:** Antimicrobials, natural products, metalloenzymes, biopharmaceuticals, biofilms

## Abstract

Natural products and analogues are a source of antibacterial drug discovery. Considering drug resistance levels emerging for antibiotics, identification of bacterial metalloenzymes and the synthesis of selective inhibitors are interesting for antibacterial agent development. Peptide nucleic acids are attractive antisense and antigene agents representing a novel strategy to target pathogens due to their unique mechanism of action. Antisense inhibition and development of antisense peptide nucleic acids is a new approach to antibacterial agents. Due to the increased resistance of biofilms to antibiotics, alternative therapeutic options are necessary. To develop antimicrobial strategies, optimised *in vitro* and *in vivo* models are needed. In vivo models to study biofilm-related respiratory infections, device-related infections: ventilator-associated pneumonia, tissue-related infections: chronic infection models based on alginate or agar beads, methods to battle biofilm-related infections are discussed. Drug delivery in case of antibacterials often is a serious issue therefore this review includes overview of drug delivery nanosystems.

## Introduction

Widespread resistance to antibiotics is now a global threat to society. Despite this the number of new antibacterials that have been brought to the market has declined considerably in recent decades. Additionally, many of the antibiotics that are in clinical development belong to existing families of compounds and their antibacterial activity and thus usefulness is likely to be decreased by rapid development of resistance in clinical strains. Therefore there is an urgent need for the development of new classes of antibacterials, preferably with new molecular targets and/or mechanism of action.

As said WHO’s Assistant Director-General for Health Security Dr. Keiji Fukuda “Without urgent, coordinated action by many stakeholders, the world is headed for a post-antibiotic era, in which common infections and minor injuries which have been treatable for decades can once again kill”. In other words, still in nearest 10–15 years there is a high risk that going to a hospital with a minor injury to die from an antibiotic resistant form of hospital infection.

Antimicrobial drug-resistant bacteria are responsible for about 33 000 human deaths annually in the European Union only. It is important to highlight that drug-resistance is growing frighteningly fast.

In this view we summarise here selected strategies for dealing with pathogenic bacteria. Since drug delivery especially in case of antibacterials often is a serious issue therefore this review also includes overview on drug delivery nanosystems.

## Application of natural products and their synthetic analogues in antibacterial drug discovery

The majority of clinically used antibiotics are natural products or derivatives thereof. The history began with the discovery of penicillin in 1928. The pool of natural products constitute a very rich source of chemical diversity, and it remains to have an untapped potential for discovery of novel antibacterial agents. According to a recent review by Newman & Cragg^1^, ca. 75% of all approved drugs in the timeframe 1981–2019 originated from or were inspired by natural products. Thus, the importance of natural products as leads for novel antibacterial agents remain evident: of FDA-approved antibacterials, 69% originate from natural products and 97% of these are isolated or derived from microbes^2^. Regardless of the success in discovery of new biologically active compounds from natural products, their use in pharmaceutical research has declined during the past decades. The main reasons for this trend stem from the lengthy process required for natural product discovery, and from the incompatibility of natural product libraries with most high-throughput screening operations. Furthermore, in many cases the originally isolated bioactive natural product is not necessarily the lead compound taken further in drug development. For example, it may not be optimal in terms of physico-chemical properties or not be available in required amounts from the original source. Alternatively, natural products may be utilised in various ways as inspiration to create biologically relevant compounds and compound collections, for example by using natural product-derived or -inspired scaffolds or fragments as starting points for synthetic libraries^3^.

Three recently developed antibiotics (i.e. eravacycline, vaborbactam and doripenem) serve as examples of utilising natural products as sources or inspiration of new antibiotics to combat antimicrobial resistance. In addition, a brief account of the discovery of anti-biofilm agents based on coniferous diterpenes (i.e. abietic acid and dehydroabietic acid) is presented.

**Scheme 1.**
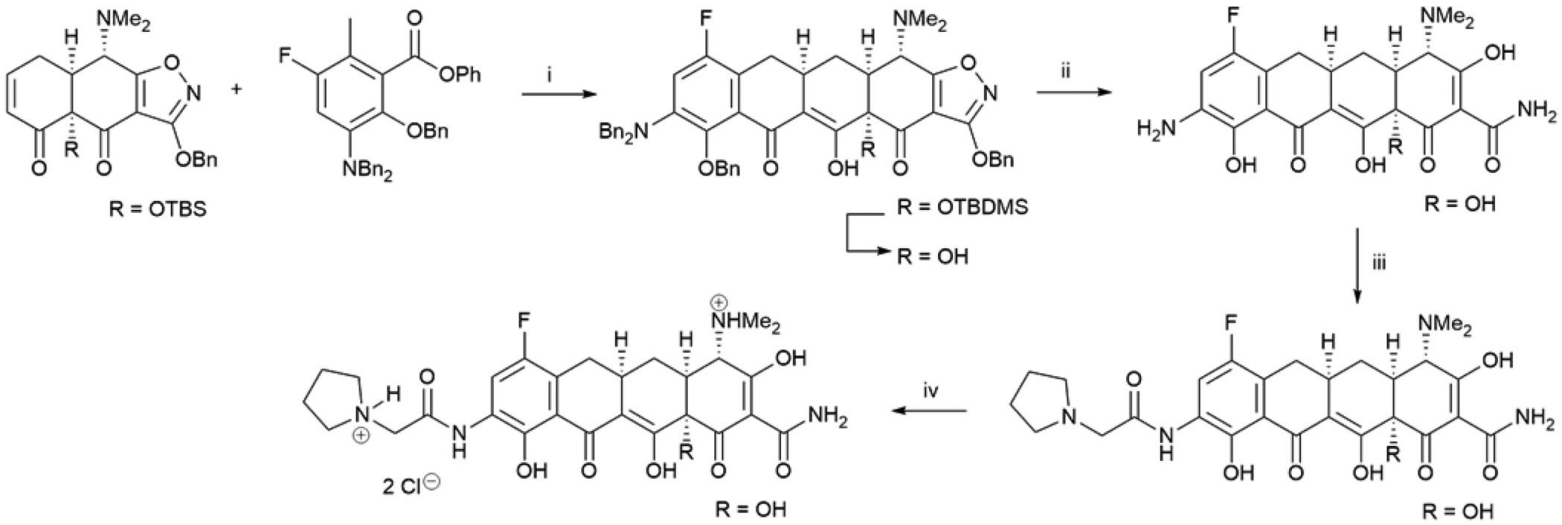
Synthesis of eravacycline. (i) LDA, TEA•HCl, THF -70 °C; LiHMDS, -70 °C to -10 °C, 94%; 48% HF, MeCN, rt; (ii) H_2_, 5% Pd-C, HCl, MeOH-H_2_O, rt, 89%; (iii) 2-(pyrrolidin-1-yl)acetyl chloride hydrochloride, MeCN-H_2_O, 10 °C, 89%; (iv) HCl, MeOH, EtOH; EtOAc, quant.

A large number of antibacterial tetracyclines, produced as secondary metabolites by the genus *Streptomyces*, have been isolated and characterised since the discovery of chlortetracycline 80 years ago^4^. The widely used tetracycline class of antibiotics has led to a reduced efficacy against pathogenic bacteria due to development of drug resistance^5^. Eravacycline (Scheme 1) is a synthetic, tetracycline-class antibiotic that has been developed for the treatment of infections caused by multidrug-resistant bacteria, e.g. methicillin-resistant *Staphylococcus aureus* (MRSA) and carbapenem-resistant *Enterobacteriaceae species*^6^. Eravacycline was approved by FDA in 2018. The four-step synthesis route of this 7-fluorotetracycline involves a tandem Michael addition and Dieckmann cyclisation as key steps (Scheme 1). This facile synthesis route also enables preparation of tetracycline libraries for further structure-activity studies.

**Scheme 2.**
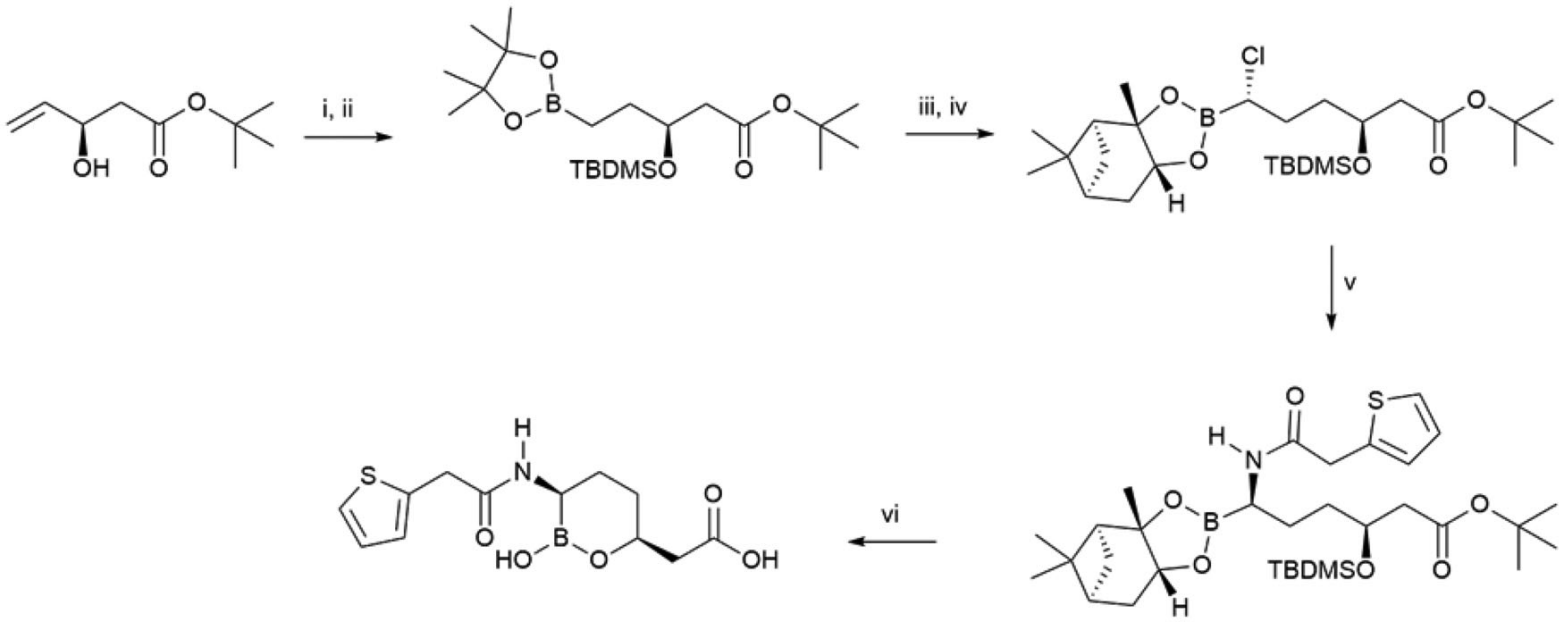
Synthesis of vaborbactam. (i) TBDMSCl, imidazole, CH_2_Cl_2_, 94%; (ii) [Ir(COD)Cl]_2_, dppb, pinacolborane, CH_2_Cl_2_, 96%; (iii) (+)-pinanediol, THF; (iv) *n*-BuLi, CH_2_Cl_2_, -95 °C, THF; (v) LiHMDS, THF, -78 °C to rt; 2-thiopheneacetic acid, EDCI, HOBT, NMM, CH_2_Cl_2_, 70%; (vi) 3 M HCl, 1,4-dioxane, Δ, 64%.

Vaborbactam (Scheme 2) is an example of a synthetic β-lactamase inhibitor that is used in combination with meropenem, an intravenous β-lactam antibiotic susceptible to degradation by metallo-β-lactamases. The FDA-approved meropenem-vaborbactam combination complements other β-lactamase inhibitors, such as clavulanic acid, a secondary metabolite of *Streptomyces clavuligerus*. The *Klebsiella pneumoniae* β-lactamase is only weakly inhibited by clavulanic acid, which therefore has no clinically relevant use against infections caused by *K. pneumoniae*^7^. Vaporbactam is a potent inhibitor of *K. pneumonia* carbapenemase with no observable concomitant off-target inhibition of mammalian serine proteases. However, vaborbactam does not inhibit class B metallo-β-lactamases, thus, further search for compounds with even wider β-lactamase spectrum is warranted. The key steps in the vaborbactam synthesis involve iridium (I)-catalyzed regioselective hydroboration of an allyl precursor, stereoselective chloromethylation of pinanediol boronate, and a stereospecific substitution of the chloro substituent with lithium hexamethylsilazide^8^.

**Scheme 3.**
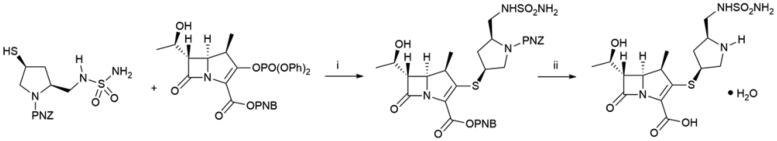
Synthesis of doripenem hydrate. (i) DIPEA, DMF, EtOAc, rt, 88%; (ii) H_2_, 10% Pd/C, MgCl_2_•6H_2_O; crystallisation; sterilisation; crystallisation, 64%.

Doripenem is a new thienamycin-inspired antibiotic, which can be used parenterally. Thienamycin, isolated from *Streptomyces cattleya* in 1976, was found highly activity against both Gram-positive and Gram-negative bacteria and is resistant to bacterial β-lactamase, however, this compound was too metabolically unstable to be further developed^9^. The thienamycin analogue doripenem has an enhanced metabolic stability to renal dehydropeptidase-1 due to the 1β-methyl substituent in the carbapenem skeleton^10^. Doripenem is a broad-spectrum antibiotic against many pathogenic bacteria, including the Gram-negative *Pseudomonas aeruginosa*. The key synthetic step (Scheme 3) is a coupling between the 4-nitrobenzyl-protected enol phosphate and substituted 3-mercaptopyrrolidine^11^.

**Figure 1.**
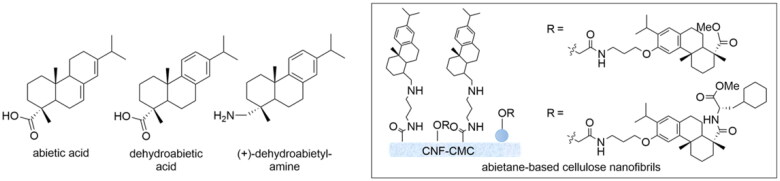
Antibacterial coniferous diterpenes and their nanocellulose surfaces.

An interesting source of antimicrobial agents is the components from resins obtained from the pines *Pinus sylvestris* L. and Norway spruce (*Piceaabies* (L.) H. Karst.). The coniferous resins have antibacterial activity against Gram-positive bacteria^12^ due to a combination of constituents, such as abietic acid and dehydroabietic acid (Figure 1). For example, various D- and L-amino acid derivatives of dehydroabietic acid disrupt *Staphylococcus aureus* biofilms and interact with the bacterial cell envelope causing efficient bacterial kill of *S. aureus* (ATCC 25923 and Newman) at low minimum inhibitory concentration (MIC 15 and 20 µM respectively)^13^.

Recently, nanocellulose-based novel biomaterials with antibacterial properties Hassan et al. have been prepared. Thus, antimicrobial nanocellulose films have been covalently bound to (+)-dehydroabietylamine^14^. These immobilised contact-active surfaces showed good antibacterial activity against methicillin-resistant *S. aureus* MRSA14TK301 and *S. aureus* ATCC12528 and were found to display excellent biocompatibility and a low risk of spreading resistance.

## Metalloenzymes as antibacterial drug targets

Around one third of all known enzymes in prokaryotes and eukaryotes are metalloenzymes^15^^,^^16^. Bacteria contain metalloenzymes, which strongly differ from those found in mammals; often metalloenzymes produced by bacteria play a crucial role for their survival^17–21^. Therefore, the identification of unique bacterial metalloenzymes and the synthesis of selective inhibitors are of great interest for the development of novel antibacterial agents^15–21^.

Among the metalloenzymes that have been investigated as potential antibacterial drug targets in some detail during the last decade are the carbonic anhydrases (CAs, EC 4.2.1.1)^22–30^. These enzymes act as highly effective catalysts for the hydration of CO_2_ to yield bicarbonate and protons, and they are involved in a multitude of physiological processes connected to pH regulation, metabolism, etc.^22–27^. In Bacteria, four of the currently eight known CA genetic families^31^^,^^32^ have been described so far, namely the α-, β-, γ- and ι-CAs^33–35^. In many species, there are CAs from more than one genetic family and frequently, more than one isoform being present. The elucidation of the role played by these enzymes during the life cycle and involvement in virulence of many pathogenic bacteria have only started recently, e.g. their inhibition proved to be bacteriostatic in many species, such as *Helicobater pylori*^36–38^, *Escherichia coli*^39^, *Mycobacterium tuberculosis*^40–43^ and more recently, also in the vancomycin-resistant enterococci (VRE)^44–46^ and *Neisseria gonorrhoeae*^47^. Indeed, for the last pathogens mentioned above, sulphonamide CA inhibitors (CAIs) such as acetazolamide (clinically used for more than 70 years) together with some recently synthesised derivatives, as well as other clinically used CAIs (e.g. dorzolamide) were recently shown to outperform linezolid, which is the drug of choice for treating VRE infections^44–46^. These promising results offer the long-awaited^44–47^ proof-of-concept for inhibition of bacterial CAs as a novel mode action for antibacterials, which are less prone to induce development of resistance. This was demonstrated for *H. pylori* and ethoxzolamide, in which case, a certain level of mutations was observed in several bacterial genes, including the α-CA one, but nevertheless the pathogen remained susceptible to the drug at sufficiently low, clinically relevant concentrations^36–38^.

Many other pathogenic bacteria besides those mentioned above encode for CAs, which may be considered as druggable targets^25–30^ – Table 1.

**Table 1.** Pathogenic bacteria and their CAs, as well as infectious disease they can cause.

Pathogen	CA class	Disease	Infectious
*Brucella suis*	*β* and *γ*	Brucellosis	contagious zoonosis (ingestion of unpasteurised milk or undercooked meat
*Vibrio cholerae*	*α*, *β* and *γ*	Cholera	severe diarrhoea.
*Helicobacter pylori*	*α*, *β* and *γ*	Gastritis and gastric ulcers	inflammation of the stomach lining, may lead to gastric cancer
*Francisella tularensis*	*β* and *γ*	Tularemia	debilitating febrile illness
*Mycobacterium tuberculosis*	*β* and *γ*	Tuberculosis	infectious attacking the lungs or other parts of the body.
*Clostridium perfringens*	*β* and *γ*	Food poisoning	death as a result of food poisoning.
*Streptococcus pneumoniae*	*β* and *γ*	Pneumonia	inflammatory condition of the lung
*Streptococcus mutans*	*β* and *γ*	Dental caries	infectious of the dental hard tissues
*Salmonella enterica*	*β*	Salmonellosis	diarrhoea, fever, vomiting, and abdominal cramps
*Haemophilus influenzae*	*β*	Influenza	nausea, vomiting, gastroenteritis
*Porphyromonas gingivalis*	*β* and *γ*	Periodontitis, rheumatoid arthritis	inflammatory diseases affecting the tissues around teeth; rheumatoid arthritis
*Legionella pneumophila*	*β*	Legionellosis	pneumonia
*Pseudomonas aeruginosa*	*β*	No particular name	infections in cystic fibrosis patients
*Escherichia coli*	*β* and *γ*	No particular name	diarrhoea (for some pathogenic strains)
*Neisseria gonorrhoeae*	*α*	gonorrhoeae	sexually transmitted disease
*Enterococcus* spp.	*β* and *γ*	No particular name	vancomycin resistant enterococci (VRE) involved in nosocomial infections

Considering the worrying levels of drug resistance emerging for the classical antibiotics by many of these and other pathogens^17^^,^^18^, targeting of these enzymes with specific and selective CAIs may lead to antibacterials with an innovative mechanism.

## Biopharmaceuticals; antisense peptide nucleic acid antibiotics

Peptide nucleic acids (PNAs) constitute attractive antisense agents that also represent a novel strategy to target bacterial pathogens due to their unique mechanism of action. PNA antisense antibiotics can in principle be developed against any protein target at the translational level by strong interactions with the corresponding mRNA (or directly against any functional RNA) due to the extremely strong PNA-RNA inter-oligomer binding observed when base pairing is optimal. Generally, PNA-based antibiotics will inherently exhibit a very narrow antibacterial spectrum, since their activity depends on the expression of a specific bacterial genetic sequence. Moreover, their activity is highly dependent on transport to the intracellular genetic target, and thus carrier efficiency is variable when targeting different species (or even strains).

Due to their inherent lack of a positive overall charge delivery across biological barriers in higher organisms is a severe challenge, which so far mainly has been overcome via conjugation to cell-penetrating peptides (CPPs). Likewise, low penetration through the outer lipopolysaccharide (LPS) layer of Gram-negative bacteria constitutes a barrier preventing efficient bacterial uptake of PNA oligomers. Thus, in order to achieve bacterial delivery of these large hydrophilic nucleic acid mimics conjugation to bacteria-penetrating peptides (BPPs) has proved to be a requisite for high potency, albeit the BBP may not necessarily contribute to the direct killing mechanism of the conjugate.

### Brief historical introduction

In most early studies focus was on targeting bacterial genes essential for maintaining metabolism (and thus survival) or bacterial replication (e.g. *acpP* involved in fatty acid biosynthesis and *ftsZ* involved in cell division). More recent work also concern genes involved in virulence, including resistance mechanisms, as recently reviewed^48–50^. Here, we will give a brief historical introduction followed by a review of selected highlights reported during the last 5 years.

The concept of antisense PNA-based antibiotics was demonstrated 15 years ago^51^^,^^52^, however, the approach remained severely limited by poor bacterial uptake of PNA oligomers due to their low propensity for translocation across membranes. The enhancer peptide (KFF)_3_K, exhibiting synergy with other antibiotics^53^ constitutes the first example of a functional BPP capable of internalising PNA as antisense PNA-BPP conjugates^52^. Later, the arginine-aminohexanoyl type eukaryotic CPPs (typically based on the RXR motif; where R = Arg and X = Ahx = 6-aminohexanoic acid) were introduced for bacterial delivery of antisense PNA oligomers as well. Studies of such conjugates, targeting essential bacterium-specific genes, provided evidence that antibacterial PNA oligomers can be designed to possess low micromolar potency against Gram-negative pathogens^54^^,^^55^. Regardless of the positive clearing effects observed in mouse infection models, these KFF-based peptides neither possess sufficient stability nor favourable toxicological properties to warrant actual drug development.

### Studies of factors influencing uptake efficiency

Optimisation of PNA-peptide conjugates targeting essential bacterial genes has largely focussed on the proper selection of target genes as well as the localisation of the antisense oligomer close to the ribosome binding site of the mRNA. Somewhat surprisingly, the PNA oligomer length optimum has been identified to be 10–12 residues, which is significantly shorter than seen for uptake by mammalian cells. Thus, the relationship between oligomer size, PNA-RNA duplex stability and antimicrobial activity in *E. coli* was recently studied in more detail^56^. Here, a direct correlation between increased oligomer length and PNA-RNA duplex stability was evident. Nevertheless, 10-mer PNAs (within a series comprising 6- to 18-mers with either (KFF)_3_K or (RXR)_4_ as delivery moieties) proved most active as antisense antimicrobials in *E. coli*, inferring an upper size constraint limiting the bacterial uptake of PNA-peptide conjugates. This was corroborated by flow cytometry experiments with fluorophore-labeled conjugates. Interestingly, the size-limited uptake appeared independent of outer-membrane integrity (which is diminished in the AS19 strain), and thus the inner membrane determines the limits in molecular size for peptide-PNA internalisation into the cytosol^56^.

Recently, the stability and uptake mechanisms as well as activity of (KFF)_3_K-eg1-PNA (and a series of analogues with shortened peptide parts) and the corresponding D-peptide analogues^57^. The SbmA inner-membrane transporter protein is known to be quite promiscuous with respect to recognition of substrates^58^, which include proline-rich AMPs and PNA-peptide conjugates. Expectedly, the L-peptide part proved to be degraded rapidly by peptidases excreted into the culture medium as well as present within the periplasm and cytoplasm. The resulting main degradation products were devoid of antibacterial activity when applied directly to the bacterial culture. Also, for both the L- and D-peptide series a gradually reduced activity was seen with decreasing length of the peptide part (i.e. MIC values changed from >16–32 µM for the naked PNA to 2–4 µM for the full-length PNA-peptide). Expectedly, the full-length D-form (kff)_3_k-eg1-PNA conjugate retained antibacterial activity against the SbmA-knockout strain, however, a surprising SbmA dependence was seen for conjugates shortened by only a few amino acids^57^. Thus, only the full-length (kff)_3_k peptide is capable of transporting PNA across both the outer and inner membrane, whereas PNA conjugates with shorter peptides (both L- and D-forms) exhibit poor spontaneous translocation across the inner membrane, and hence require SbmA-mediated transport for efficient cytoplasmic uptake, and thus antibacterial activity.

One of the latest studies on peptide conjugates with PNA (H-CTCATACTCT-NH_2_), targeting the *acpP* gene expression in *E. coli*, concerns how the composition of LPS affects their bacterial uptake, and thus their antibacterial activity^59^. In Gram-negative bacteria the outer membrane mainly consists of O-antigens attached to outer-core sugars (comprising 5 different monosaccharides in *E. coli*) linked to inner-core sugars, which are linked to lipid A. The inner core sugars comprise at least two Kdo (3-deoxy-D-manno-oct-2-ulosonic acid) residues and one or more L-glycero-D-manno-heptose (Hep) residues, and is highly conserved within a species. Firstly, *E. coli* MG1655 (which is devoid of O-antigen) was compared with derived strains having partially or fully restored O-antigen. Interestingly, the O-antigen part appeared to exert limited effects on the activity of (KFF)_3_K-eg1-PNA, (RXR)_4_X-(β-Ala)-PNA, and (RX)_6_-(β-Ala)-PNA (X = aminohexanoic acid; eg1 = ethylene glycol linker) as similar MIC values were obtained for both strains^59^. Intriguingly, the O-antigen-restored strain exhibited faster time-kill kinetics than the wild type (MG1655) when treated with either (KFF)_3_K-eg1-PNA or (RXR)_4_X-(β-Ala)-PNA, and hence the O-antigen part in this case may act as an apparent binding site, while for other compounds (e.g. AMPs) it is believed to prevent efficient penetration of the envelope. In addition, four inner-core mutants with increasingly shortened LPS core were found to be significantly (8- to 32-fold) more susceptible to the (KFF)_3_K-eg1-PNA (MICs in the range 0.03–0.12 µM) than the parent strain (MIC of 1 µM). By contrast, these mutants displayed similar susceptibility to the two Arg-rich conjugates as that of the parent strain, inferring that the outer membrane is not the rate-limiting barrier for the uptake of such conjugates. Incomplete LPS inner core enables improved access to the periplasm, and thus conjugates may more readily reach the inner membrane containing the SbmA transporter essential for the (KFF)_3_K-PNAs^59^. Thus, these findings corroborate the hypothesis that uptake and activity of SbmA-transported (KFF)_3_K-PNA mainly is limited by translocation across the outer membrane, whereas uptake and activity of the SbmA-independent RXR-PNA type conjugates mainly is limited by inner membrane translocation.

### PNA-peptide antimicrobials against Pseudomonas aeruginosa

Discovery of novel antimicrobials against *P. aeruginosa,* capable of inhibiting bacterial growth as well as the ensuing inflammatory response, constitutes a key goal in research aimed at alleviating progression of cystic fibrosis. Interestingly, targeting the translation initiation region of the essential *acpP* gene in *P. aeruginosa* by a known growth-inhibiting (RX)_6_B-PNA conjugate^54^ was found to exert a concomitant strong inhibition of expression of pro-inflammatory chemokines and cytokines (i.e. IL-8, IL-6, G-CSF, IFN-γ, IP-10, MCP-1 and TNF-α)in IB3-1 cystic fibrosis cells infected by *P. aeruginosa* PAO1. In particular, the attenuation of IL-8 induction is significant due to its key role in the exacerbated pro-inflammatory cellular response induced by *P. aeruginosa* infection in cystic fibrosis patients^60^.

### AMPs as delivery vehicles in PNA conjugates

Rapid resistance development against PNA-peptide conjugates has been found to be associated with mutations in the non-essential gene *sbmA*^55^, encoding an inner-membrane ABC transporter, for which proline-rich peptides previously has been identified as typical substrates^54^^,^^55^. Hence, novel carriers should preferably act via an SbmA-independent uptake mechanism, and such peptide carriers have been identified^55^^,^^61^. A recent alternative approach for achieving efficient bacterial delivery of PNA-peptide conjugates that has been explored is the use of antimicrobial peptides (AMPs) attached to the antisense PNA oligomer to provide dual-action antibiotics^61^^,^^62^.

In particular AMPs with an intracellular mode of action were found to constitute promising vehicles for bacterial delivery of an antibacterial PNA oligomer targeting the essential *acpP* gene in *E. coli.* Thus, it was demonstrated that buforin2-A (BF2-A), drosocin, oncocin, Pep-1-K, and KLW-9,13-a as well as the end-modified BF-2A-RXR and drosocin-RXR (X = aminohexanoic acid) indeed are capable of transporting PNA effectively into *E. coli* (with resulting MICs of 1–4 µM). Importantly, the presence of the inner-membrane peptide transporter SbmA was not required (cf. test in ΔsbmA versus wild-type strains) for antibacterial activity of PNA-AMP conjugates containing Pep-1-K, KLW-9,13-a, or drosocin-RXR. Consequently, certain AMPs (and modified analogues thereof) provide efficient transport of PNA into the bacterial cytoplasm with the AMP moiety appearing to act simultaneously at a separate target (e.g. the ribosome)^61^]. Similarly, N-terminally modified drosocin (i.e. RXR-PRPYSPRPTSHPRPIRV; X = aminohexanoic acid, Ahx) and a truncated Pip1 peptide (i.e. [RXR]_2_-IKILFQNRRMKWKK; X = Ahx) were subsequently studied. The drosocin-derived compound without a linker moiety exhibited the highest antibacterial activity against both wild-type *E. coli* and *K. pneumoniae* (MICs in the range 0.3–0.7 µM), while analogues displaying an ethylene glycol (eg1) moiety or a polar maleimide linker were found also to exhibit activity towards wild-type *K. pneumoniae* (MICs of 0.6–1.3 µM). Against two colistin-resistant *E. coli* strains the linker-deficient compound proved most potent (with MICs in the range 0.3–0.7 µM). Importantly, the antisense PNA-drosocin conjugates had potent antibacterial activity against colistin- and tigecycline-resistant *E. coli* and *K. pneumoniae* without exhibiting concomitant haemolytic properties^62^.

### Cationic dendrons for delivery of PNA oligomers

Besides Arg-based modification of AMPs to obtain more efficient delivery vehicles for PNA, a recent work reports on guanidinylated dendrimeric delivery moieties for PNA targeting the translation of the acpP mRNA essential for fatty acid synthesis^63^. Based on a series of 2,4-diaminobutanoic acid (Dab)-based dendrons, the structure-activity relationships (SARs) were explored with respect to the effects of terminal guanidinylation as well as the length (and thus hydrophobicity) of the terminal linear cationic carbon linker (C_4_ to C_8_) moieties. Expectedly, the compounds with terminal amino groups had low activity towards *E. coli* (i.e. MICs above 16 µM). Conversely, the corresponding guanidinylated derivatives exhibited MIC values in the ranges 0.25–2 µM and 0.125–8 µM against *E. coli* and *K. pneumonia*, respectively. The C_8_ linker conferred highest activity, however, the mismatch PNA conjugate also possessed substantial activity (MICs of 4–8 µM) that appeared to arise from membrane-disruptive effects^63^. To assess the influence of the size of the core dendritic structure on antibacterial activity, two similar third-generation dendrons with a core of 2,3-diaminopropanopic (Dap) residues were examined as well. However, only slight differences in activity between the Dap- and Dab-based conjugates were observed, implying that the outer shell of the dendron is most important in determining the antibacterial properties of dendron-PNA conjugates. The guanidinobutanoyl-modified Dab-based dendron with a charge of +8 was subjected to further studies, which showed: (i) SbmA-independent activity, (ii) low cytotoxicity against the liver cell line HepG2 (full viability at 90 µM), (iii) MICs increased in the presence of high concentrations of divalent cations (Ca^2+^ and Mg^2+^), (iv) bactericidal mode of action, (v) high stability in mouse and human serum, with t_1/2_ ≫24 h, (vi) *in vivo* activity against a multidrug-resistant *E. coli* in a murine peritonitis model^63^.

### PNA-peptide antimicrobials against Klebsiella and Acinetobacter species

In another recent study, a PNA conjugate with the (KFF)_3_K CPP targeting *gyrA* in KPC-producing *K. pneumoniae* was found to inhibit bacterial growth *in vitro*. The suppressive effect on the expression of the *gyrA* gene was evaluated along with the haemolytic properties towards mouse erythrocytes. The conjugate was capable of inhibiting bacterial growth at 50 µM, with a concomitant reduction of the 16S gene amplification by ca. 97%, while 21% haemolysis was observed at this concentration^64^.

Nosocomial infections caused by carbapenem-resistant *Acinetobacter baumannii* (CRAB) are emerging as increasingly difficult-to-prevent and -treat diseases. A series of BPP-PNA oligomers targeting the translation initiation region of the *ftsZ*, *acpP*, and *rne* genes of CRAB strains, comprising the antisense conjugates (KFF)_3_K-eg1-(*acpP*)PNA, (KFF)_3_K-eg1-(*ftsZ*)PNA, and (KFF)_3_K-eg1-(*rne*)PNA, were found to exhibit complete growth inhibition against several CRAB strains and isolates at concentrations in the range 1–2 µM, while the compounds were bactericidal either at the MIC or at 2-fold higher concentrations. Expectedly, the mode of killing did not involve a concomitant membrane disruption. In addition, the conjugates exhibited low cellular toxicity in the HepG2 cell line (up to 20 µM) and did not show significant antibacterial activity against other Gram-negative pathogens (i.e. *E. coli* and *P. aeruginosa*), indicating a potential for specific treatment of CRAB infections^65^.

### PNA-peptide antimicrobials against other gram-negative pathogens

Also, other Gram-negative pathogens have been considered as potential targets for PNA-based conjugates. For example, six PNA-peptide conjugates, displaying (RFF)_3_R at one of the PNA termini, were designed to target the expression of the gene for the acyl carrier protein (*acpP*) in non-typeable *Haemophilus influenzae* (NTHi), and these displayed MICs in the range 0.6–2.5 µM against 20 clinical isolates. Biofilm eradication (as measured by biofilm-eradicating concentrations = MBECs) was only seen at significantly higher concentrations (i.e. MBECs up to 40 µM). Resistant strains could be selected by serial passages upon initial exposure to sub-MIC concentrations that were gradually increased. A resistant strain had an SNP predicted to affect an ATP-binding protein of a conserved ABC transporter. Interestingly, insertion of an ethylene glycol linker (eg1) between PNA and delivery peptide affected the propensity for resistance development, since the wild-type strain remained susceptible to this conjugate even after 30 serial passages^66^.

In addition, PNA conjugates targeting two Gram-negative pathogens, critically associated with periodontitis, have been designed and evaluated as antisense antibiotics for selective growth inhibition of *Porphyromonas gingivalis* and *Aggregatibacter actinomycetemcomitans*. Antisense PNAs targeting *groEL* or *acpP* were conjugated to the traditional delivery peptide (KFF)_3_K. In *P. gingivalis* the anti-*groEL* PNA conjugate inhibited growth for 5 h at a concentration of 3 µM. Anti-*groEL* PNA against *A. actinomycetemcomitans* inhibited growth for 2 h at a concentration of 3 µM, with an ensuing reduced GroEL protein expression. In contrast, anti-*acpP* PNA exhibited no marked growth-inhibitory effect on these species. Thus, anti-*groEL* PNA-peptides appear to constitute potential species-specific antibacterial tools against oral pathogens^67^.

In a comparative study of KFF-PNA, RXR-PNA and Tat-PNA anti-*acpP* conjugates [i.e. (KFF)_3_K-, (RXR)_4_XB-, and GRKKKRRQRRRYK-ctcatactct], global RNA-seq analysis was used for the first time to investigate how the transcriptome in *Salmonella enterica* is affected upon exposure to the same PNA oligomer when delivered by different carrier peptides^68^. Most previous studies have focussed on MIC determinations to assess activity of peptide conjugates with PNA oligomers designed to prevent ribosomal interaction with the target mRNA, however, the peptide part may trigger pathways protecting the integrity of the bacterial envelope. Indeed, besides inhibition of protein synthesis, a rapid *acpP* mRNA decay occurred, the exact mechanism of which remains to be elucidated. Intriguingly, conjugates with different carrier peptides did not only exhibit different bactericidal activity (MICs in the range 1.25–5 µM, with KFF-PNA being the most potent), they also triggered *acpP*-independent stress pathways. In particular, KFF-, RXR- and Tat-PNA conjugates all induced the PhoP/Q response, while the last two also induced upregulation of gene expression related to several membrane proteins and transporters.

### Alternative targets for PNA-based antimicrobials

Intriguingly, alternative targets for PNA-based antimicrobials have been explored with a successful outcome. Thus, the bacterial signal recognition particle (SRP) was considered as a potential antibacterial target, for which proof of principle was achieved by using an antisense (KFF)_3_K-PNA conjugate to target a key SRP-related RNA-protein interaction. In liquid culture, one PNA conjugate inhibited the growth of *E. coli* AS19 cells in a dose-dependent manner with complete growth inhibition at a concentration of 2.5 µM. Transmission electron microscopy showed that deformed cell structures were seen for PNA-treated bacteria. Depending on the targeted RNA site, this strategy may be tailored either to generate broad-spectrum PNA-based antibacterial compounds, or to identify sequence-specific inhibitors for drug-resistant pathogens^69^.

Bacterial toxin–antitoxin (TA) systems comprise genetic modules that encode a growth-arresting protein toxin (acting by interference with essential cellular processes), and a cognate antitoxin, which neutralises the activity of the toxin. TA systems have no human homologs, but they are abundant in bacterial genomes, and they thus constitute attractive alternative antibacterial targets. Artificial activation of *E. coli* mazEF and hipBA toxin–antitoxin systems was explored as an innovative approach, in which arrest of growth in *E. coli* was induced via inhibition of translation of the antitoxins by antisense (KFF)_3_K-PNA oligomers. The MIC of the anti-*mazE* PNA conjugate was 16 µM in three different *E. coli* strains, while the anti-*hipB* PNA inhibited growth of *E. coli* K-12 and WR3551/98 with MICs of 8 and 16 µM, respectively. Furthermore, the combination of anti-*thyA* PNA with trimethoprim resulted in a highly synergistic interaction (FICI = 0.31; for the combination 4 µM PNA + 0.04 µM antibiotic). These findings infer that TA systems constitute interesting, but so far poorly explored targets for antisense agents^70^.

Plasmid-mediated drug resistance is accelerating the spreading of polymyxin resistance, resulting in only a few or no remaining therapeutic options for treatment of infections caused by certain Gram-negative MDR bacteria (e.g. carbapenemase-producing strains). In order to restore susceptibility to colistin in *Enterobacteriaceae*, PNAs designed to specifically target *mcr-1* expression were explored^71^. In particular, a 12-mer PNA-peptide conjugate [AACTACTCAAAA-O-(KFF)_3_K] targeting a mRNA sequence (at the ribosome-binding site that is conserved in nine *mcr-1* variants) proved capable of complete inhibition of the expression of *mcr-1* at a concentration of 4 µM, thereby conferring an increased susceptibility to colistin (with a MIC decreasing from 8 to 2 µg/mL). Importantly, 4 µM of this PNA-peptide conjugate improved colistin susceptibility in several *mcr-1*-positive *E. coli* strains (35 out of 37 tested) with MICs decreasing from 4–16 µg/mL to 1–2 µg/mL. Encouragingly, this strategy may increase the lifespan of the critical polymyxin antibiotics, albeit the toxicity and other potential side effects of such PNA conjugates require further *in vivo* studies to assert their safety as preclinical drug candidates.

Ribonucleotide reductases (RNRs) constitute the rate-limiting enzymes involved in *de novo* synthesis of DNA precursors via reduction of ribonucleotide di-/tri-phosphates to deoxyribonucleotides di-/tri-phosphates. *Escherichia coli* possesses genes encoding an iron-dependent class Ia RNR, which is essential for viability during aerobic growth (i.e. nrdAB; with nrdA and nrdB transcribed as a polycistronic mRNA with the nrdA gene located upstream of the nrdB gene). A PNA-peptide conjugate ([KFF]_3_K-eg1-ATGTATGTCG-NH_2_) was designed to target *E. coli nrdA*, and this anti-NrdA-PNA indeed inhibited bacterial growth with MIC value of 4 µM^72^. In addition, during treatment DNA synthesis rate was found to decrease, resulting in incomplete chromosome replication. It is proposed that reduced DNA replication is caused by dNTP depletion, which eventually causes an accumulation of double-stranded DNA breaks. Importantly, anti-NrdA-PNA treatment did not influence protein synthesis, corroborating the intended mode of action^72^.

### PNA-peptide antimicrobials against gram-positive pathogens

Recent examples of a PNA-based antibacterial strategy against Gram-positive pathogens have also been reported. *Streptococcus pyogenes are* a human pathogen causing a wide range of infections that usually can be treated with penicillins, albeit cases of therapeutic failure have emerged. Also, streptococcal resistance to alternative antibiotics (e.g. macrolides) is common. Previously, the HIV-1 Tat peptide coupled to anti-*gyrA* PNA was shown to inhibit growth of *S. pyogenes*, but later an investigation of 18 CPP-coupled anti-*gyrA* PNAs on *S. pyogenes* was undertaken^73^. Here, HIV-1 Tat, oligolysine (K_8_), and (RXR)_4_XB (X = Ahx; B = β-Ala) conjugates with anti-*gyrA* PNAs were found to inhibit bacterial growth *in vitro.* Treatment with these three PNA-CPPs led to increased survival of larvae in a *Galleria mellonella* infection model^73^.

Nosocomial infections caused by antibiotic-resistant *Enterococcus faecalis* have increased prevalence, and thus novel strategies are needed. The *efaA* gene (presumed to be involved in biofilm formation) was targeted by an antisense (RXR)_4_-PNA conjugate, which was designed to interact with the start codon section of the *efaA* gene. Biofilm inhibitory effect of the conjugate was seen at 10 µM, where expression of the *efaA* gene was essentially abolished, while biofilm formation was reduced by 60% after 24 h treatment. In addition, in an MTT assay the conjugate was devoid of toxicity on MCF7 cells at the active concentration^74^.

### A novel transport mode for PNA-peptide antimicrobials

A novel transport mode for antibacterial antisense PNA oligomers was recently explored. Besides CPPs and BPPs new types of bacterial delivery vehicles for antibacterial PNAs should be considered, and the vitamin B12-PNA conjugates indeed constitutes a novel approach for enhancing bacterial uptake of PNA oligomers. Thus, vitamin B12 was covalently linked to a PNA oligomer targeting the mRNA of the *acpP* gene in *E. coli* either via a stable linkage or via a cleavable disulfide linkage. The MICs of vitamin B12 − PNA conjugates against *E. coli* were 5 µM, similar to that of the corresponding (KFF)_3_K-PNA control. Moreover, vitamin B12-PNA conjugates proved to be stable in the presence of biological matrices^75^.

### Combination therapies: synergy and sensitisation

A combination of antibacterial agents is considered to make the emergence of resistance in bacteria less probable. Thus, potential synergy between antibacterial antisense PNA-peptide conjugates and conventional antibiotics was tested in the *E. coli* AS19 (lipopolysaccharide defective) strain and in a modified pathogenic strain *E. coli* O157:H7. Herein, PNAs were designed to target *acpP* (i.e. anti-*acpP* PNA) and conjugated to (KFF)_3_K. Antibiotics studied included aminoglycosides, aminopenicillins, polymyxins, rifamycins, sulphonamides and trimethoprim. Two novel synergistic combinations were identified, namely (KFF)_3_K-PNA and polymyxin B as well as (KFF)_3_K-PNA and trimethoprim - both with a fractional inhibitory concentration index (FICI) of 0.38. Synergy with polymyxin B indicates that antibiotics impairing the integrity of the bacterial envelope may be attractive agents for improving uptake of PNA conjugates, whereas trimethoprim/PNA-peptide synergy may arise via a hitherto unknown mechanism^76^.

Likewise, the *lpxB* gene in *A. baumannii* was evaluated as a potential therapeutic target for antisense PNAs either alone or in combination with known antimicrobial therapies. RNA-seq analysis of *A. baumannii* ATCC 17978 showed that the *lpxB* gene was overexpressed in a murine pneumonia model. Expression *in vivo* of LpxB during treatment with anti-*lpxB* (KFF)_3_K-PNA was inhibited *in vitro*, and gave rise to decreased bacterial survival while increasing the survival rate of infected A549 cells. Synergy was observed between this conjugate and colistin in colistin-susceptible strains. Test via an *in vivo Galleria mellonella* infection model confirmed that combination treatment with anti-*lpxB* (KFF)_3_K-PNA and colistin was more effective than colistin monotherapy^77^.

Most recently, a larger study on the potential of PNA-peptide conjugates as sensitisers for antibiotics in wild-type *E. coli* and *P. aeruginosa* was reported^78^. Here, PNA oligomers were designed to silence gene expression of *bamB* and *tolC*, coding for a component of the outer membrane protein assembly complex (β-barrel-assembly machinery) and TolC (part of the substrate-promiscuous AcrAB-TolC efflux pump complex), respectively. Firstly, KFF-bamB [(KFF)_3_K-eg1-catcgggtcc] and KFF-tolC [(KFF)_3_K-eg1-tgcattcctt] at low concentrations (i.e. 5 µM) were shown not to exert significant growth inhibition alone (but at high concentrations they caused a slight growth delay). Interestingly, KFF-bamB decreased the MICs in *E. coli* of novobiocin, and fusidic acid by 16-, and 4-fold, respectively, while the combination of KFF-bamB and KFF-tolC decreased the MIC of these antibiotics even more pronounced (>1000-fold to below 0.4 µg/mL). To expand this sensitisation approach to *P. aeruginosa*, conjugates targeting bamB and oprM (a tolC homolog) were designed: RXR-bamB [(RXR)_4_-XB-catatcattg] and RXR-oprM [(RXR)_4_-XB-tcaggcctct]. These were applied at a concentration of 3 µM together with antibiotics that were inactive alone (vancomycin, erythromycin and carbenicillin). In the presence of RXR-bamB carbenicillin MIC was reduced from 64 to 8 µg/mL), while the combination (1:1) of RXR-bamB and RXR-oprM reduced the MICs of carbenicillin and erythromycin from 64 and 128 µg/mL to below 4 and 16 µg/mL, respectively. This constitutes the first report on the potential of bamB as a target of antisense PNA-based conjugates, and the increased effect when applied in combination with conjugates targeting oprM indicates that it will be possible to design sensitiser cocktails to combat various pathogens with different resistance mechanisms.

Another approach for enhancing activity of antibiotics is the combination therapy involving membrane-permeabilizing compounds. In this context the polymyxin-derived NAB741^79^ (displaying a neutral Ac-Thr-D-Ser tail instead of the C_8_/C_9_ fatty acyl-Dab-Thr-Dab with two cationic charges; Dab = 2,4-(S)-diaminobutanoic acid), was at a sub-MIC concentration (1.6 µg/mL) found to provide >10-fold improved antibacterial activity of (KFF)_3_K-eg1-PNA in wild-type *E. coli* (MG 1655 strain)^57^. Interestingly, the effect was even more pronounced for conjugates with shortened peptide parts, e.g. the activity was enhanced 32-fold for the KFFK-eg1-PNA (i.e. a reduction in MIC from 16 to 0.5 µM). This large enhancement of antibacterial activity by NAB741 infers that transport across the outer membrane constitutes the main barrier when SbmA is present as an efficient inner-membrane transporter. In contrast, only a 2- to 4-fold enhancing effect of NAB741 was found for the D-form KFF-based conjugates, indicating a different mode of outer-membrane passage, a less efficient inner-membrane translocation by SbmA, or an alternative mechanism for crossing the inner membrane^57^.

### Conclusive remarks

Conclusively, antisense PNA-peptide conjugates constitute a unique compound class amenable to rapid drug discovery as medicinal chemistry on the active principle can be performed without significantly affecting its affinity for the therapeutic target (i.e. typically a species-specific mRNA involved in a process essential for bacterial survival). We and others have characterised the drug potential of PNA-peptide conjugates, of which several have been tested in mouse infection models. However, further optimisation towards lower toxicity and increased efficacy in *in vivo* settings is clearly warranted, and for these reasons no antibacterial agents belonging to this compound class have as yet entered clinical trials.

## *In vivo* models to study biofilm-related respiratory infections

Bacteria can grow as single cells (planktonic growth state) or as a sessile community, also known as a biofilm. According to the National Institutes of Health (NIH), biofilms are involved in over 80% of all bacterial infectious diseases. Due to the increased resistance of biofilms to antibiotics, alternative therapeutic options are urgently needed.

To develop novel antimicrobial strategies, standardised and optimised *in vitro* and *in vivo* models are needed. To bridge the gap between *in vitro* findings and clinical relevance, animal models are required^80^. These models differ in genetics, physiology and biochemistry, but are used under controlled and ideal lab circumstances, thereby minimising variables such as sex, age and environment^81^^,^^82^.

While it is clear abandoning all animal experiments for the sake of animal welfare is impossible, as both computational and experimental *in vitro* and *ex vivo* models cannot consider all variables and individual variations essential to an *in vivo* situation, efforts to reduce their need are being made^83^. Alternative *in vivo* models are gaining attention as these models are often more simplistic compared to complex vertebrate models. They are not able to fully replace animal experimentation but could be an extra step in- between *in vitro* and *in vivo* experiments, leading to a reduction of laboratory animals^84–86^. Invertebrate models such as insects and fish are commonly used in the academic area, especially in the larval stage^87^.

Although important information has been generated using *in vitro* and invertebrate models, the use of mammalian models that are more closely related to humans is required for going from bench to bedside. It is challenging to mimic BRI in higher organisms, but this challenge has to be tackled in order to address diagnostic, preventive or therapeutic solutions^88^.

### *In vivo* biofilm models

Non-human mammalians, i.e. mice and rats, are mostly chosen to unravel the *in vivo* pathogenesis of bacterial lung infections or to evaluate the *in vivo* efficacy of new antibacterial therapies or prophylactic agents. The use of mice as a laboratory animal species has many advantages, including the availability of a wide arrange of molecular and immunology laboratory techniques specific for mice, the easy housing and care and the requirement of low quantities of test compounds due to the small size of mice making it a cost-efficient model^89^. Extrapolation of results obtained from mouse lung infection models to humans is not straightforward due to significant anatomical and physiological differences between mice and human. Despite the differences in lung anatomy and respiratory parameters between mouse and human, similarities are present when comparing pneumonia in both species. One important similarity is the accumulation of the polymorph exudate in the alveoli of pneumonia patients as wells as in murine lungs infected with bacteria.

Differences between the murine and human lungs are well documented, such as differences in structural anatomy, tracheobronchial epithelium, local phagocytic and chemical defences and immune response^87^^,^^90^. It is important to consider those differences when extrapolating data from *in vivo* experiments. The murine lungs exhibit a monopodial branching pattern as opposed to the symmetric branching pattern in human lungs. Murine lungs have therefore fewer respiratory bronchioles than human lungs^91^. The airways of mice terminate abruptly into alveolar ducts without intervening to respiratory bronchioles^92^^,^^93^. The cellular composition of the murine tracheobronchial epithelium differs due to the presence of less mucous and serous cells and the absence of submucosal glands^94–96^. Some characteristics of the antimicrobial molecules, that are a part of the innate lung defense, differ as well. Differences in local phagocytic defences and cellular expression and ligand binding for some toll like receptors were also observed for both species^97^^,^^98^.

Chronic pneumonia models can be used to study biofilm formation of *P. aeruginosa* as well as the influence of single virulence factors or to study co-infection with other pathogens (e.g. *B. cenocepacia* or *S. aureus*)^87^^,^^99–107^. The *P. aeruginosa* lung infection in these chronic models can be challenged with antibacterial and anti-biofilm therapies to evaluate their efficacy^108–114^. Disease models such as CF knock-out mouse models were also developed to study *P. aeruginosa* or other bacterial lung infections. Models of *P. aeruginosa* lung infection can be divided by their route of infection in intratracheal or intravenous models^88^. Intratracheal models represent the clinical situation better. A relevant model was developed by Cash et al., 1979^115^ using immobilised bacteria in agar beads. Pedersen et al., 1990^116^ developed another model by embedding the bacteria in seaweed alginate microspheres.

### Device related infections: ventilator-associated pneumonia (VAP)

Hospitalised patients are at high risk of morbidity and even mortality from healthcare- associated infections (HAIs), in addition to their primary illness. According to the ECDC, 8.9 million European patients acquire infections in hospitals and long-term care facilities every year^117^. HAIs cause over 90,000 deaths per year in the European countries^118^, more than any other infectious disease under surveillance. And hospital-acquired pneumonia is the second most common nosocomial infection and the most common cause of death from HAI in critically ill patients^119^. Ventilator-associated pneumonia (VAP) is a serious hospital-acquired pneumonia which is defined as lung infection acquired by patients mechanically ventilated in the intensive care unit for at least 48 h^120^. Patients with VAP have increased length of hospitalisation and consequently increased health cost^121^^,^^122^.

Endotracheal tube microbial colonisation is a major risk factor for VAP and a multispecies biofilm of commensal micro-organisms forms in 90% of the tubes used in mechanical ventilation^123^. This biofilm presents a clinical challenge since pathogens can attach to the microbial community formed on the inner surface of the endotracheal tube. Carried by airway pressure, they can then bypass normal barriers and reach the pulmonary parenchyma or infect the lungs. In a prospective observational study that included patients from 27 European intensive care units (ICUs), *S. aureus* is reported as the most common Gram-positive isolate in VAP patients and *P. aeruginosa*, *A. baumannii* and *E. coli* are among the most common Gram-negative isolates^124^. Mortality rates can vary in different patient groups and depend on underlying conditions and disease severity. In a meta-analysis using randomised trials and considering a homogeneous group of patients, the overall attributable mortality associated with VAP was 13%^125^.

To address this high risk of mortality from VAP, several strategies have been investigated such as modifying endotracheal tubes in order to make their surface anti-fouling and/or prevent biofilm formation by incorporating substances with antibacterial properties during the manufacturing process^126^. The limited number of available solutions is due to the complex process of developing reliable devices, starting from idea up to the market. For a successful development, an interdisciplinary approach is essential, requiring drug development, material science, engineering, clinically relevant animal models and, finally, well-designed clinical studies. Thus, the availability of tools to produce testing samples for each step of characterisation and validation is key to achieve the required results.

Several animal models have been developed to study preventive or curative strategies for VAP, using mice, sheep, pigs or dogs. They all rely on tracheal intubation of the animal with endotracheal tubes^88^^,^^127^. Each animal model has its own advantages and limitations. The benefits of using a mouse model are their small size and relatively rapid reproductive rate. There are also genetic and physiological similarities to humans and genetically engineered mutant mice strains, such as CF mouse models^128^^,^^129^. The costs of a mouse model are limited. Disadvantages are that the anatomy and physiology of their respiratory tracts differ from humans. For instance, mice have no respiratory bronchioles and a weak cough reflex. Other limitations are long-term mechanical ventilation is unfeasible, there is limited ability to provide critical care to severely ill animals and, therefore, using mouse experiments to predict the long-term effectiveness and safety in humans remain controversial^130^.

Murine models of VAP can be divided regarding the necessity or not of surgery (tracheotomy) and the possibility or not of mechanical ventilation. A research group at Nagasaki University has been using endotracheal intubation in mice as the preferred non-invasive procedure to study VAP treatment options against *P. aeruginosa*^131–134^. However, this model uses tubes with shorter length and direct infection of the lungs and does not include the possibility of studying the effects of the biofilm formation on the endotracheal tube. Surgery has been used not only in mice but also in rat models, to study the effects of inhalation therapy with or without simultaneous mechanical ventilation^135–137^. However, the time of the experiment is shortened to 4 h maximum when ventilation is performed. To study this aspect and mimic VAP appropriately other mammal models such as sheep, pigs and dogs must be employed, since only after 48 h ventilation the models would resemble the clinical situation^138–141^. Most models test a monospecies infection with *P. aeruginosa*, the most frequent pathogen associated with VAP^121^^,^^142^.

Shaqour et al. developed a test system for evaluating novel antimicrobial compounds in a VAP mouse model. Therefore, the tubes produced with this novel design were loaded with ciprofloxacin (CPX), a well-known antibiotic for treating *S. aureus* VAP. The advantages of incorporating CPX in the tubes are its thermal stability and low cost. From a clinical perspective, CPX should not be used prophylactically, since it could be used for treatment of VAP caused by Gram-negative bacteria^143^. The tubes were not cytotoxic, and their antibacterial properties were demonstrated by zone-of-inhibition (ZOI) and quantification of the planktonic bacteria. Their antibiofilm properties were also confirmed by quantification and visualisation of the attached bacteria.

Animal models of device-related infections can be divided in site-specific and subcutaneous models. The VAP mouse model is a site-specific model and allows the evaluation of the host response to endotracheal tubes located in the same position as in the clinic^88^. Other animal species have been used to study BRIs in endotracheal tubes such as sheep, pigs and dogs^138^^,^^140^^,^^141^. Mice have the advantages of their small size, relatively high reproductive rate and the availability of antibodies, increasing the chance to bring new technologies and drugs from bench to bedside. We used fully mature adult mice to ensure that the tubes fit in the trachea. Outbred SWISS-CD1 are used in all areas of biomedical research and are more resistant to infections than the inbred mice as reported in a *P. aeruginosa* VAP model^144^.

The challenge to establish a VAP mouse model lies not only in the technical, but also in the biological aspects. The selection of a bacterial strain is fundamental for the success of murine models^88^. The benefit of this animal model is the relative ease to establish a chronic infection (≥ 3 days)^127^^,^^145^ with *S. aureus* ATCC 25923. The limitation is that the delivery of bacteria at implantation of the device is initially different between the CPX and the TPU tubes, since the bacterial viable numbers are reduced during the pre-incubation step due to eluting CPX. However, in the clinical situation an endotracheal tube is placed in the trachea in contact with the fluid lining of the tracheal epithelium. In this humid environment, CPX elution will also start at the same time as the first contaminating bacteria will reach the tube. Thus, it might be argued that the exposure of the bacteria to CPX in the pre-incubation step represents the situation that may occur *in vivo*. In future studies, it would be interesting to focus on the pathophysiology by inserting non-colonized tubes and subsequently exposing mice to the bacteria. Other bacterial strains and even species could be used for this purpose, such as *P. aeruginosa*, since chronicity of infection has been previously obtained with the appropriate strain and methodology^107^^,^^131^.

### Tissue-related infections: chronic infection models based on alginate or agar beads

Mice are inherently more resistant to infections caused by human bacterial lung pathogens. Two methods can be used to overcome this problem. Immunosuppression, i.e. suppression of the cell-mediated immunity, mostly by PMNs, makes mice more susceptible to human bacterial lung pathogens^146^. However, considering the high virulence of the bacteria used in this study immunosuppressing the mice would further compromise their well-being. An alternative for immunosuppression is embedding bacteria in beads followed by pulmonary infection^147^. Bacteria are embedded in an immobilising extracellular matrix formed by agar, agarose or seaweed alginate^99^^,^^101^^,^^115^^,^^116^. This embedding technique results often in a chronic lung infection as bacteria are protected from the host immune system inside the beads instead of being quickly removed from the lungs. Chronic *P. aeruginosa* lung infections are characterised by the presence of protective biofilm structures. This adaptive strategy of *P. aeruginosa* contributes to their persistence by avoidance of the immune system or by hindering the activity of antibacterial therapies. Cash and collaborators developed a chronic *P. aeruginosa* lung infection mouse model by embedding bacteria in agar beads following administration to lungs of rats^115^. The use of agar beads bypass the primary host airway defense mechanisms such as the interaction between bacterial adhesion molecules and respiratory epithelial cells. In this way, the initial colonisation phase of *P. aeruginosa* is avoided. The use of these *P. aeruginosa* agar beads resulted in retention of bacteria in the airways leading to a persistent stimulation of the host immune system which is typical for CF lungs. A disadvantage of this model is that the extensive neutrophil influx in response to the *P. aeruginosa* agar beads may cause airway obstruction and consequently lead to ineffective gas-exchange. A small percentage of animals may die after the infection procedure^148^. An advantage is that with this model lung infection can last more than one month in lungs of infected rats and cause inflammation and tissue damage that is similar to the one observed in CF patients^115^. Pedersen and collaborators adapted this chronic lung infection model by using seaweed alginate beads instead of agar beads^116^. A major advantage of the use of seaweed alginate is to mimic closely the natural CF lung conditions, in which mucoid *P. aeruginosa* are present in biofilms and the extracellular matrix of these biofilms consist mainly of pseudomonal alginate^116^^,^^149^. This model was modified by Starke et al., 1987 for Swiss mice^101^ and optimised to cause chronic infection in BALB/c mice by Moser et al., 2009^107^.

## Methods to battle biofilm-related infections

### Quorum sensing inhibitors

It is well known that growth of bacteria in biofilms can lead to increased resistance against antimicrobial therapies, caused by the decreased growth rate of the bacteria, the inaccessibility of the bacteria inside the biofilm and the possible presence of persister cells. For these reasons antibiotic therapy failure is commonly seen in BRI’s^150^. Therefore, novel strategies need to be developed to battle biofilm-formation.

An important new target is the bacterial communication system, quorum sensing (QS). The QS-system consists of extracellular signal molecules that are produced and secreted by bacterial cells to coordinate their behaviour in a cell-density dependent manner and thereby synchronously altering their gene expression. The main QS-mechanism used in gram-negative bacteria is the production of acyl homoserine lactone (AHL) signalling molecules^151^. Although the involvement of QS in biofilm formation is not yet fully elucidated, quorum sensing inhibitors (QSI) have been proposed as promising anti-biofilm agents. Several methods of QS inhibition have been proposed including the inhibition of signal synthesis or degradation of the signal, receptor inhibition or directly interfering with the downstream signalling cascade^152^^,^^153^.

Compounds interfering with signal synthesis mostly relate to analogs of S-adenosylmethionine (SAM) which is the amino donor for the formation of the homoserine lactone ring moiety. These include S-adenosyl homocysteine, sinefungin and butyryl-SAM^154^^,^^155^. Also, the antibiotic azithromycin has been shown to interact with the C_4_-homoserine lactone synthesis leading to reduced adherence to polystyrene surfaces^129^^,^^156^.

The main target of the inhibition of the QS-signalling complex is the enzymatic degradation of the signal molecule also called Quorum Quenching (QQ), including AHL-lactonase and -acylase as well as oxidising and reducing agents like paraoxonase and oxidoreductases. These are all responsible for the breakdown of AHL leading to reduced accumulation and biofilm formation by the sessile bacteria^153^^,^^157–160^.

A third method in inhibiting QS is receptor inhibition of mostly the LuxR-homolog receptor. This was done by using AHL-analogs that match this receptor. They mostly differ from AHL by replacing the lactone ring by a cyclopentyl or cyclohexanone ring. In this way, a signal-receptor complex is generated that is competitive to the native active receptor-complex leading to a disruption of the downstream signalling cascade^161–163^. Besides these, also unrelated compounds resembling AHL are used that can be found in nature. This includes bergamottin (component of grapefruit juice), extracts of *Penicillium* species (patulin) and garlic extracts (ajoene)^164–167^.

### Microbiome-based therapy

Due to increasing treatment failure because of antibiotic resistance and the unfavourable cytotoxic properties of QS-inhibitors different treatment strategies have been investigated in the past decade. One of the strongest candidates is the use of probiotics in the battle against biofilm-infections^168^. Probiotics can be used to reduce the adherence of pathogenic bacteria to surfaces as well as hinder their activity^169^^,^^170^. Besides that, they also interact with QS-mechanisms, biofilm formation and survival of pathogenic bacteria leading to a reduced biofilm-quality and ultimately eradication of the bacterial biofilm. Mechanisms used by the probiotics include the creation of unfavourable conditions (pH alterations, surface and nutrient competition) and the production of antagonists (e.g. surfactants, enzymes,…)^171–174^. These agonists lead to disruption of the bacterial membrane, altered gene expression in pathogenic biofilms and modulation of the host immune response to the biofilms^175–177^. All of the above mechanisms have been proven to be methods employed by different probiotic bacteria including *Lactobacillus spp.,* some *Streptococcus* species like *S. salivarius* and *S. oralis* and *Bifidobacterium lactis* and *B. longum*^168^. Cell free supernatants or membrane vesicles from probiotic strains can be used alone or in combination with the probiotic bacterial cells as an innovative antimicrobial formulation^178^.

Another bacterium that is associated with the Cystic Fibrosis (CF) respiratory tract is *Rothia mucilaginosa*. Research has pointed out that *Rothia*-species exhibit an anti-inflammatory effect in CF-lungs as has been shown in both *in vitro* and *in vivo* models by Rigauts et al. Besides that, in a cohort of patients with bronchiectasis, a negative correlation has been observed between the presence of *Rothia* and the expression of pro-inflammatory markers (IL-8, IL-1β) in the sputum. The proposed mechanism by which *Rothia* exhibits its function is the inhibition of the NF-κB pathway activation by reducing the phosphorylation of IκB-α leading to a decreased expression of NF-κB target genes^179^.

## Antibiotic drug delivery nanosystems

The inappropriate use of antibiotics and new bacterial defence mechanisms has led to a substantial decrease in the efficacy of traditional antibiotics. The high number of antimicrobial resistance (AMR) cases against key antimicrobial groups represent a serious threat to patient safety in Europe^180^. Therefore, it is essential to develop new approaches for killing bacteria and to avoid development of AMR^181^. Development of new drug delivery systems is a powerful tool to enable alternative mechanisms for systemic administration and for a controlled release of a drug^182^. These systems comprise vesicular systems, such as liposomes or niosomes. Extracellular vesicles (EV) have also a potential as drug delivery systems due to their role in intercellular communication and in transfer of biological information^183^.

Nanotechnology and tissue engineering have been utilised in the elaboration of nanoparticle (NP) based antimicrobial agents. Some of the potential advantages of NPs in the fight against microorganisms are their low propensity to generate resistance, and they constitute a safe alternative to facilitate clinical use of otherwise difficult-to-administer drugs^184^^,^^185^

Encapsulation of an antibiotic in a niosomal formulation may enhance its antibacterial efficiency. Due to the compatibility of the physico-chemical properties between antibiotics and vesicles encapsulation of traditional antibiotics in nanovesicles may be achievable^186^.

**Figure 2.**
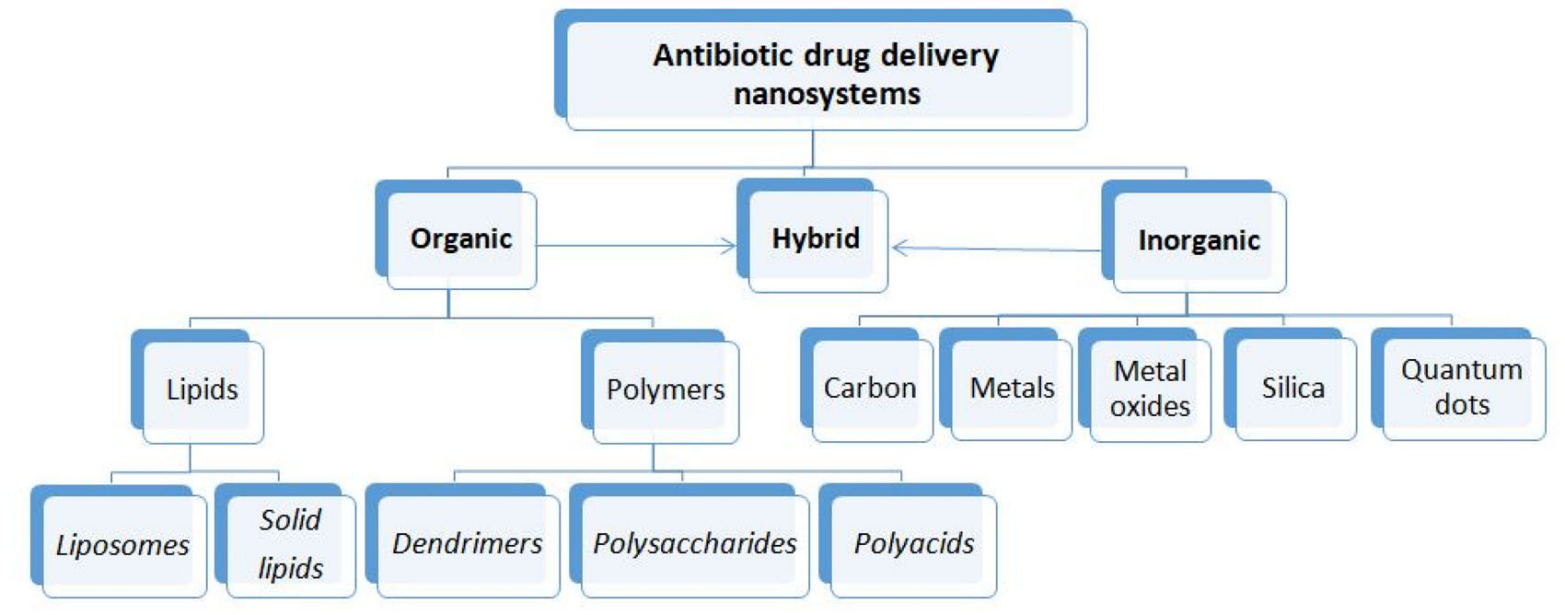
The nanoparticulate drug delivery systems being explored in antimicrobial therapy.

In the last decades development of antibiotic drug delivery systems can be divided into two main directions: (i) evaluation of different kinds of nanovesicular systems and (ii) their applications. There are different types of nanoformulations available for antibacterial applications. Mainly nanoparticulate drug delivery systems formed by either an organic or inorganic core, or hybrid nanosystems consisting of both components are may be used for these applications^187–190^. The NPs classes being explored in antimicrobial chemotherapy are summarised in Figure 2.

### Antibiotic delivery systems on inorganic material core

Inorganic nanosystems represent a class of nanosystems derived from inorganic materials – metals, metaloxides, carbon materials, silica nanoparticles and quantum dots^189^^,^^190^

Metallic NPs generally composed of Au, Ag, or Cu have been shown to possess strong antimicrobial activity^191^. Furthermore, because of the small size of the NPs, they facilitate transport of active substances across biological barriers, and facilitate release of the drug in the proper site(s)^192^. The use of metal NPs as drug carriers is one of the recent rapidly developing approaches in chemotherapy^193^. Metal NPs as drug carriers appear beneficial due to their ability to solve problems of solubility of hydrophobic drugs, and increase the dissolution efficiency of drug molecules in the blood^194^. Because of considerable interactions between the particles and the medium, agglomeration may occur. Moreover, metallic NPs on their own may induce harmful effects, in particular to healthy cells^194^. Antimicrobial activities of metals and metaloxides have been widely studied. Highly potent antibacterial effects have been shown by a number of metaloxide NPs, which include silver (Ag), iron oxide (Fe_3_O_4_), titanium oxide (TiO_2_), copper oxide (CuO), and zinc oxide (ZnO). Most metaloxide NPs exhibit bactericidal properties through generation of reactive oxygen species (ROS), while some are effective due to their physical structure and release of metal ions^195^.

#### Silver nanoparticles

Silver exhibits favourable effects because of its chemical stability, excellent conductivity, catalytic, and antibacterial activity. Nowadays, silver nanoparticles (AgNPs) are among of the most widely studied^196^^,^^197^. It is known that AgNPs are effective antimicrobial agents against bacteria, fungi, and viruses^198^^,^^199^. In medicinal use, Ag-containing compounds are found useful in treatment of burns, wounds, and various infectious diseases^200^^,^^201^. The antimicrobial potency of AgNPs, like other metals or metaloxide NPs, has been found^202^. Despite the fact that the mechanism of action for AgNPs remain unclear, AgNPs with small diameters have been found to possess higher antimicrobial potency than those with larger diameters^203^. Silver is also known as an efficient bactericidal agent that can kill various pathogens *in vitro* and *in vivo*^204^. Furthermore, bacteria appears to have a lower propensity for development of resistance against Ag than seen for ordinary antibiotics^205–207^.

Nevertheless, there is numerous aspects that remain to be addressed: silver minimal inhibitory concentration (MIC), minimum bactericidal concentration (MBC) clinical breakpoint, effects on emergence of resistance^208^^,^^209^, nanoparticle toxicity, and side effects of AgNPs^210^^,^^211^. Importantly, the details in the bactericidal mechanism of AgNPs remains to be elucidated^212^.

Usually, particles with diameters below 100 nm are suitable for drug delivery applications, and AgNPs that meet this requirement are readily obtained. Coating of NPs with active substances, may considerably increase the size of the resulting modified particles, and therefore it is crucial that the core itself is small. The accumulation of AgNPs at disease site depends on a facilitated translocation across membranes, which is better for small-size AgNPs. However, smaller particles may increase intracellular cytotoxicity caused by their degradation. For neutral AgNPs cytotoxicity should be lower than for drug-loaded particles. The attractive force among the conducting electrons of AgNPs and bacterial cell membranes facilitate their adhesion to the surface, thereby launching a toxic effect. AgNPs, having a diameter less than 50 nm, change the potential of bacterial cells quite efficiently and prevent their proliferation^213^. AgNPs also demonstrate synergistic antibacterial effects in combination with different antibiotics on both Gram-positive and Gram-negative bacteria^214^^,^^215^.

AgNPs are effective antimicrobials, and constitute promising alternatives to traditional antibiotics. The evaluation of potency of differently coated 10 and 50 nm-sized AgNPs against Gram-negative *E. coli* and Gram-positive *S. aureus* demonstrated that 10 nm-sized AgNPs released more Ag ions and were more toxic than 50 nm-sized AgNPs, while coating-dependent activity was more prominent for 50 nm-sized AgNPs^216^. Recently, novel silver ultra-nano clusters were synthesised with an average size less than 5 nm^217^. These clusters were effective in eradicating *H. pylori* biofilms and showed only a low toxicity on human gastric adenocarcinoma cells.

The exact mechanism of action of AgNPs is still not clear. Mechanistically, the antimicrobial profile of AgNPs is correlated to their attraction to bacterial surface, destabilisation of bacterial cell wall, change in membrane permeability, induction of toxicity and oxidative stress by generation of reactive oxygen species (ROS) and free radicals as well as modulation of signal transduction pathways^218^^,^^219^.

Several new silver‐based antimicrobial products have been commercialised in the last two decades^220^ and a recent study investigated surgical sutures coated with titanium dioxide nanoparticles covalently linked with monovalent silver ions for its potential prophylactic activity against surgical site infections^221^.

#### Gold nanoparticles

Gold nanoparticles (AuNPs) are colloidal or clustered particles comprised of an inert and biocompatible gold core^222^. One of the advantages of AuNPs is their synthetic versatility allowing for control of their size, shape and surface properties. To enable conjugation of biologically active molecules the NP surface can be modified with thiols or amines, thereby providing functionalised AuNPs^223^. Despite the potential applicability of AuNPs, some uncertainty remains about their potential toxicity^219^. AuNPs are active against Gram-negative and Gram-positive bacteria, namely *E. coli, P. aeruginosa, S. typhimurium, Serratia spp., K. pneumoniae, S. aureus, B. subtilis* and *E. faecalis,* among others^224–226^ In comparison to Ag, AuNPs show lower antibacterial effect by themselves^212^. The adsorption of active drug molecules on the AuNPs surface allows for delivery of active ingredients to targeted sites. Various antimicrobial agents such as antibiotics, antimicrobial peptides, and surfactants can be conjugated onto Au shells to confer bactericidal activity^227^. Regardless of the scope of application of AuNPs, it is very important to maintain their stability by preventing their aggregation^194^.

#### Copper and copper oxide nanoparticles

Copper is a semiconductor material considered to be an excellent candidate for the synthesis of copper based NPs (CuNPs). Along with being highly resistant to heat, it is robust, stable, cheap, and can be readily obtained^228^^,^^229^. Although copper oxide (Cu_2_ONPs and CuONPs) are effective against various bacterial pathogens, their antibacterial efficacy is somewhat lower than that of Ag or ZnO (see below). Thus, a relatively higher concentration of NPs is needed to achieve the same level of activity^230^. In addition, the activity CuONPs varies considerably depending on which bacterial species is to be targeted. Nevertheless, as Cu is less costly than other metal nanomaterials, it can be used as nanocomposite to enhance the efficacy of antibiotics.

CuONPs also possess antimicrobial effects in themselves. The mechanism of antibacterial activity of CuONPs is as yet not well clarified, but it is assumed that it involves bacterial cell wall adhesion caused by electrostatic interactions. Released Cu^2+^ions induces generation of ROS that interact with cellular membranes. The ROS is also capable of penetrating the cell, causing bacterial cell membrane damage, which is associated with degradation of the internal contents and bacterial cell leakage^231^. Moreover, their antibacterial activity has been studied in detail against microorganisms such as *E. coli*, *V. cholera*, *P. aeruginosa*, *S. typhus*, *S. aureus*, *E. faecalis*, *B. subtilis* and *S. faecalis*^219^^,^^232^^,^^233^.

#### Zinc oxide nanoparticles

Due to photocatalytic properties of zinc oxide nanoparticles (ZnONP) shave the ability to generate ROS quite readily, which gives rise to favourable antibacterial properties. ZnONPs have a positive surface charge, and therefore they will readily interact with negatively charged bacterial membranes, which facilitate penetration into the cells, leading to their destruction. These features allow them to be applied as excellent additives for dentistry bactericides and daily cosmetics products. ZnONPs have an isoelectric point at pH ∼9. At pH values below 9, ZnONPs are surface-protonated to the form of ZnOH^2+^, which means that they remain positively charged in the pH range corresponding to body fluids, which promotes their applicability as drug carriers^234^. Photoluminescent features, used in biosensing, together with antibacterial properties, are promising parameters for the putative use of these NPs in theranostics. Use of ZnO in self-lighting photodynamic therapy (SLPDT) against human ovarian cancer (OVCAR-3) cells in culture was described a decade ago. It was proven that under dark conditions, the ZnONPs conjugated with methylthioadenosine phosphorylase (MTAP) anticancer drug to safely reach its intended site, and when exposed to ultraviolet A (UVA) radiation they were able to produce ROS, which significantly reduced the viability of cancer cells. Cells died both by apoptosis and necrosis^235^. Those studies produced a clear evidence that use of ZnONPs in combination with already known anticancer drugs may enhance the effect of cancer therapy^194^.

#### Titanic oxide nanoparticles

Also, titanium dioxide (TiO_2_) has been extensively studied for its antimicrobial activities^236^. TiO_2_ is known for its ability to kill both Gram-positive and Gram-negative bacteria^237^. More recently it was found that titanic oxide nanoparticles (TiO_2_NPs) were effective against various viral species and parasites^195^^,^^238^.

TiO_2_ are characterised by a refractive index in the UV/visible range. TiO_2_, like ZnO, is an n-type semiconductor material^239^. TiO_2_NPs are relatively inexpensive, highly chemically stable, non-toxic, biocompatible, and also highly active. Depending on the parameters and the method used, it is possible to control the shape and size of the particles. A number of methods are currently available for the preparation of TiO_2_NPs. Among them, the most used are sol-gel, hydro, and solvothermal methods along with electrochemical methods^240^. The controlled release of daunorubicin (DNR) was observed when using TiO_2_ NPs as a drug carrier. This release was observed due to the possibility to change pH. It was noted that lowering the pH from 7.4 to 5.0 accelerated the release of DNR from TiO_2_NPs^241^.

The combinations of Ti or TiO_2_ with other NPs such as AgNPs were found to have a synergistic effect and to enhance their activity^242–245^.

#### Superparamagnetic iron oxide nanoparticles

Iron oxide NPs have found wide applications in different fields of biomedicine^246^. The antibacterial properties are described both for NPs based on iron oxides and as free iron ions. However, in contrast to free ions, iron oxides do not exert a significant toxic effect on mammalian cells^247^^,^^248^. Among iron oxide NPs, superparamagnetic iron oxide nanoparticles (SPIONs) consisting of Fe_2_O_3_ or Fe_3_O_4_ being the most important ones. They are mainly used as contrast agents in imaging (MRI), but they have also found applications as targeted drug carriers and biosensors. Toxicity studies of SPIONs show that they may be mutagenic and neurotoxic, can induce genotoxicity, and also cause adverse effects when subjected to long-term exposure^194^^,^^239^. Microbiological assays have proven that surfaces modified by Fe_3_O_4_ nanoparticles demonstrate anti-adherent properties, and therefore significantly reduce both Gram-negative and Gram-positive bacterial colonisation^195^^,^^249^. Comparative studies of the antibacterial properties of ferrimagnetic and superparamagnetic NPs led to the conclusion that both nanomaterials may be considered as antibacterial candidates for treatment of complicated infections in the upper respiratory tract or in the skin^250^. SPIONs can be coated with other NPs such as Ag and Au, and their magnetic properties can be utilised to penetrate and destroy biofilms^251^^,^^252^ or to enable surface modification with other materials, for example oleic acid, alginate or polyethylene glycol to prevent *P. aeruginosa* biofilm formation^253^. It has been suggested that the capping agent plays a major role in conferring bactericidal properties to such nanocomposites. Alternatively, several surface modifications of SPIONs were used for optimisation of the activity and increasing their interaction with the bacterial cells^254^. These NPs can be modified with chemical groups and metals can be adhered to them. As the example, synthesis of silver-ring-coated SPIONs involves the coating of monodispersed SPIONs with carboxylated dextran via the ligand exchange followed by conjugation with ethanediylbis(isonicotinate)^255^. Also conventional immobilisation of amphiphilic 1,4-dihydropyridine (1,4-DHP) on aminosilane-coated magnetic NPs markedly enhances their antimicrobial activity as compared to non-immobilised molecules due to the higher affinity of these nanosystems for bacterial cell wall components^256^. Good combination of the antimicrobial activity and biocompatibility of iron nanoparticles makes them particularly attractive study objects as antibiotic carriers^257^.

#### Carbon-based nanomaterials

During the past years carbon-based NPs have been used for antiviral applications. Usually, carbon nanomaterials exert low cytotoxicity and exhibit specific antiviral activities. Carbon-based nanomaterials have yielded encouraging results and may provide the necessary level of biocompatibility and antiviral properties. The main groups of carbon nanomaterials explored as antiviral systems comprise e.g. fullerenes, carbon nanotubes (CNTs), and in particular single-walled carbon nanotubes (SWCNTs) as well as graphene oxide (GO) NPs^258^. Due to a high surface/volume ratio and other unique chemical and physical properties of carbon-based nanostructures the study of their antimicrobial properties is particularly interesting. Furthermore, application of functionalised carbon nanomaterials as delivery systems of antibiotics may reduce the associated resistance, improve bioavailability and facilitate targeted delivery^259^. The size of carbon NPs is of importance for the inactivation of the microorganisms. Thus, shorter tubes demonstrate more effective bactericidal performance in comparison to longer tubes. CNT-cell interactions in a liquid medium are rather different if compared to the solid surface. Tube diameter is also significant for the bacterial inactivation process. Tubes with smaller diameters can provide higher damage to cell membrane though the cell-surface interaction^260^^,^^261^. Direct contact with microorganisms is their main mechanism of action, which involves interference with their cellular membrane integrity, metabolic processes and morphology^258^^,^^259^.

Structural features such as type of core and surface functionalities of carbon dots (CDs) have a crucial influence on their interaction with other cells and organisms, and this can be used in the design of probes to target specific bacterial classes. Due to their fluorescent properties CDs are used to label and image bacteria. CDs have also found applications as photosensitisers, which provide excellent spatiotemporal control upon irradiation with light of the production of ROS leading to membrane damage. Nanocomposite CDs containing antimicrobial agents including antibiotics and transition metals are effective theranostic agents displaying synergistically improved antimicrobial activities. Incorporation of CDs in hydrogels, nanofibrous materials, and polymers is one of the ways for improvement of materials for application in wound healing and regeneration, and also of materials with a long-lasting protection against infections^262^.

#### Silica nanoparticles

Mesoporous nanostructures are attractive building blocks due to their biomolecule adsorption/desorption capabilities^263^^,^^264^. Mesoporous silica-based nanoparticles (MSiNPs) offer advantages in nanotechnology because of their applicability in the design of complex systems and their cost-effectiveness. Their specific surface characteristics, porosity and possibilities for functionalization make them useful in therapeutic delivery^265^. MSiNPs constitute a material, which combine an efficient drug delivery profile and facile (bio)chemical modification^266–268^. The antibacterial effect of silicon-based nanocoatings was demonstrated on Gram-positive (*S. aureus*) and Gram-negative (*P. aeruginosa*) bacteria by using them in the form of biofilm-durable polymeric matrices^269^. The modification of particles with antibiotics was one of the first nanotechnological strategies explored to combat bacterial infections^268^. Various antimicrobials such as antibiotics, peptides, and other functional materials can be covalently or non-covalently loaded onto MSiNPs, offering a rational solution to the issues of resistance, physiological barriers and functional barriers such as drug solubility or toxicity^270^.

#### Quantum dots

Quantum Dots (QD) are a type of fluorescent nanomaterial developed recently. QDs are tiny particles or nanocrystals of a semiconducting material with diameters within the range 2–10 nm. QDs are semiconductor nanocrystals with peculiar photoluminescence properties. Use of QDs for biological imaging is one of the most successful new nanobiotechnology developments^260^. These particles are comprised of a semiconductor inorganic core such as CdSe and coated shell such as ZnS improving optical properties and solubility in aqueous buffers^271^. Polymer-functionalised QDs possessing more promising features and higher antibacterial activity have been reported^272^. Functionalised QDs could be applied in antibacterial research as an effective alternative to traditional antibiotic drugs due to their exceptional antibacterial mechanisms. Functionalised QDs can insert into the cell membrane, thereby destroying the cell barrier and produce a large amount of ROS, thereby damaging the cell components^273^. Compared with other functionalised NPs, QDs with quantum size are generally more effective against microorganisms than metal-based NPs with sizes ranging within 30–100 nm^274^. Additionally it has been demonstrated that heavy metal-free QDs selectively eliminate multidrug-resistant (MDR) pathogenic bacteria, while remaining non-toxic to human host cells^275^. CdTe-QD are non-toxic at concentrations relevant for reducing the bacterial load of MDR *E. coli* infections in a murine subcutaneous abscess model. These QDs destroy bacteria through the generation of superoxide^276^.

### Antibiotic delivery systems based on cores of organic materials

Organic nanosystems-liposomes, lipid-based NPs, polymeric NPs have preferable biodegradability and biocompatibility features, making them suitable as candidates for clinical use^190^^,^^277^.

#### Lipid-based nanoformulations

Lipid-based nanoformulations such as liposomes, nanoemulsions, and solid lipid NPs (SLNs), are often employed as delivery systems for antibacterial drugs. Liposomes can easily bind to bacterial membrane, and deliver antibiotics directly to bacteria^278^.

Liposomes are concentric, bilayered vesicles formed by a membranous lipid bilayer, which mainly is composed of natural or synthetic lipids. Liposome diameters are within the size range of 0.01–5.0 µm, and they can act as a carriers for a variety of drugs^279^^,^^280^. The antibacterial activity and the pharmacokinetics properties of antibiotics can be enhanced by entrapping them within liposomes. Use of lipid vesicles as drug carriers may influence drug distribution and reduce toxic effects^281^. Liposomes are usually non-toxic, biodegradable and can encapsulate both hydrophobic and hydrophilic drugs without any chemical modification. In addition, liposomes can be composed of materials possessing distinctive desirable properties such as long systemic circulation time, specific cell targeting, and release that is sensitive to pH, reductive environment and/or temperature^282^. Another outstanding feature of liposomes is their ability to fuse easily with the bacterial membranes, thereby releasing the drug within the cell membrane, or into the interior of the microorganism^283–285^. As a result, the effective dose of the drug and its toxicity decreases^286^. Additionally liposomes protect the entrapped drug against enzymatic degradation before reaching its target. For example, liposomes protect penicillins and cephalosporins from degradation by β-lactamases, which are produced by certain microorganisms^287^^,^^288^. Recently, several reviews on antibiotic encapsulation in liposomes, comprised of various lipids, have been published^289–292^.

During the last years attention has been dedicated to the development of stimuli-responsive nanocarriers, such us pH-, enzyme-, redox-, and ionic-microenvironment-dependent antibacterial therapy^293–295^. It was also found out, that liposomes may be designed to fuse with bacterial cells, holding the potential to overcome antimicrobial resistance and biofilm formation and constituting a promising solution for the treatment of potential fatal MDR bacterial infections e.g. caused by methicillin-resistant *S. aureus*^278^^,^^292^^,^^296^.

Another way to improve the activity of liposomal antibiotic system is to promote the synergy between drug and lipid activity. It has been found that various natural and synthetic lipids used for liposome preparation also possess inherent antibacterial activity. For example, it was demonstrated that selected fatty acids and cholesteryl esters packaged with phospholipids possessed antibacterial activity against Gram-positive and Gram-negative bacteria, and thus may augment the activity of antibiotics demonstrating a potential as novel lipophilic antimicrobial agents^297^. The toxicity of the 4-(*N*-alkylpyridinium)-1,4-DHP derivatives on both Gram-positive and Gram-negative bacteria species and eukaryotic microorganisms has been studied, and the results showed that activity depended on the alkyl chain length at the *N*-alkyl pyridinium moiety and the number of propargyl groups. These lipid-like compounds warrant further exploration as constituents of drug formulations^298^. Also sulfonium-based antibacterial lipids for the delivery of antibiotics have been recently developed. The presence of cationic sulfonium moieties and the inherent membrane-targeting properties of the lipids contributed to the reduction of antibiotic resistance development in bacteria, and also enabled delivery of antibiotics to remove the infectious pathogens selectively. Sulfonium-based liposome-encapsulated antibiotics is an example of synergy between drug and lipid^299^.

Solid lipid nanoparticles (SLNs) are the most effective lipid-based colloidal delivery systems, introduced in the end of the 20th century. In contrast to liposomes, SLNs do not have a two-layer structure and are composed of a solid fat matrix and stabilised by surfactants. The main benefits of SLNs are their long-term stability, ease of use, encapsulation of hydrophilic and lipophilic drugs, and biocompatibility^300^. SLNs are within the size range of 50–1000 nm, and they are composed of physiologically tolerated lipid components, which are in the solid state at room temperature^301^. For preparation of SLNs solid lipids such as stearic acid, glyceryl monostearate, glyceryl distearate, and decanoic acid as well as surfactants such as soy lecithin, egg lecithin, sodium cholate, sodium glycocholate, tween 20 and 80, or polaxamers 188, 182, or 407 are used^302^. Proposed advantages of SLNs include the possibility of controlled drug release, high drug payload, excellent bioavailability, avoidance of the use of organic solvent, no toxicity of the carrier, easy upscaling and sterilisation, increased drug stability, and possibility for incorporation of lipophilic and hydrophilic drugs^303^.

#### Polymeric nanoparticles

Some polymers also exhibit antimicrobial properties in themselves. Such antimicrobial polymeric materials can be employed for biomedical devices and in healthcare to avoid the resistance problems associated with use of antibiotics^304^. Polymeric particles produced from natural and/or synthetic polymers are biocompatibile and often multifunctional, and they may also possess high stability *in vitro* and *in vivo*. The polymeric particles allow for controlled and sustained release of antibiotics in order to improve antibacterial efficacy against biofilm-related infectious diseases^305^. The commonly used natural polymers are human or bovine serum albumin, gelatine, collagen, alginate, chitosan, and starch. Use of synthetic polymers such as poly (α-hydroxy acids), polyanhydrides, polylactide, poly(lactide-co-glycolide) (PLGA), and poly(fatty acid dimer-sebacic acid) has been reported also^306^.

Dendrimers are hyperbranched polymers with a precise nanoarchitecture and low polydispersity, which are synthesised in a layer-by-layer fashion around a core unit, resulting in a high-level control of size, branching points, and surface functionality. The highly dense surface of functional groups allow for the synthesis of dendrimers with specific and high binding affinities to a wide variety of viral and bacterial receptors^280^.

The encapsulation of antibiotics in dendrimeric systems can improve their therapeutic efficacy and minimise their side effects. A primary point in the design of dendrimers as delivery systems are the control of particle size, the properties of the surface, the functionality and branch length/density, and the proper release of drugs to obtain the desired effect on the targeted site^307^. Drugs, including antibiotics, can be trapped inside dendrimers, be physically adsorbed onto their surface or chemically attached to the dendrimer surface. Encapsulation of antibiotics in dendrimeric materials is a tool for an improvement of their pharmacokinetic and pharmacodynamic properties^308^. Both hydrophilic and hydrophobic agents can be loaded at the same time either by encapsulating drug within the dendrimer structure, or by interacting with the drugs at their terminal groups via electrostatic attacation, or via covalent linkage to suitable functional groups^187^.

One of the most studied dendrimers for the release of antibacterial drugs is the PAMAM dendrimer. PAMAM is a non-immunogenic and biocompatible compound that is water-soluble^309^. PAMAM materials are regarded as carriers or drugs (e.g. antibacterials or antifungals), which have a capacity to improve solubility, drug permeation, and therapeutic efficiency^309^. Based on the type of linkage and chemical moieties presented in the molecule, dendrimers can be divided into four types: glycol dendrimers^310^, peptide dendrimers^311^, and Janus dendrimers^312^^,^^313^.

Chitosan is obtained by deacylation of the natural polymer chitin. Properties such as biocompatibility, biodegradability, and also inherent broad-spectrum antimicrobial properties make chitosan suitable as a carrier for antibiofilm agents^314^. Chitosan NPs were found to be effective against 24-hold *P. aeruginosa* biofilms of six different clinical isolates^315^.

The surface charge of chitosan NPs is positive, allowing them to interact with negatively charged bacterial cell walls and biofilm-derived extracellular polymeric substance (EPS) surfaces^316^. Chitosan derivatives possess the antibacterial activity, which depends on the type of microorganism, the Mw or degree of deacylation of chitosan, the concentration of chitosan, pH value of media and temperature^317^. On one hand biocompatibility, degradability, and antimicrobial nature of chitosan constitute the features, which make it suitable for antibiotic delivery into biofilms, on the other hand the hydrophobicity of chitosan promotes aggregation under biological condition, and hence limits its use. Aggregation of chitosan NPs could be observed after an hour in cell medium^318^. The addition of poly(ethylene glycol) (PEG) to the surface of chitosan NPs proved to be an efficient way to overcome this obstracle^319^. NPs of chitosan modified with linoleic acid were used for immobilisation of the enzyme β-N-acetylglucosaminidase (DspB), which then was found to be more effective as compared to the free enzyme with respect to inhibition and detachment of biofilms via degradation of poly-β(1,6)-N-acetyl-glucosamine (PNAG), a major polysaccharide found in the EPS produced by *S. epidermidis*, *S. aureus*, and *Actinobacillus actinomycetemcomitans*^320^.

Alginate is biopolymer derived from the cell wall of algae, and it is commonly used for construction of nanocarriers for drug delivery. Alginate is a naturally occurring polysaccharide, which unlike chitosan, is an anionic polymer. Alginate-based NPs can be loaded with antimicrobial agents for treatment of tuberculosis or fungal infections^321^. Usage of alginate has several advantages including ease of preparation, biocompatibility, biodegradability, and absence of toxicity^322^. Alginate-based beads were reported to be useful for sustaining the release of poorly water-soluble flavones^323^.

Poly(lactic-co-glycolic acid) nanoparticles (PLGA NPs). Poly(lactic-co-glycolic acid) (PLGA) is a biocompatible and degradable copolymer. The ester linkages in PLGA are degraded through hydrolysis in the presence of water, and the polymer degradation products lactic acid and glycolic acid are natural byproducts of several metabolic pathways. Various drug delivery devices made of PLGA NPs are FDA approved^316^. Generally, the size of PLGA NPs is within the range 100–400 nm^324^. PLGA NPs provide robust drug delivery systems. Most PLGA-encapsulated antibiotics show a biphasic sustained release profile *in vitro*. With the surface decoration of PLGA NPs with PEG, the biocompatibility of PLGA NPs can further be improved, and the circulation time elongated when the application necessitates this. The main disadvantage of PLGA NPs is low encapsulation efficiency, especially of hydrophilic agents^316^.

Poly-*ε*-lysine. Poly-*ε*-lysine is a cationic homo-oligopeptide of L-lysine found to be active against Gram-positive and Gram-negative bacteria. It also exhibits activity against the spore forms of *B. coagulans, B. stearothermophilus and B. subtilis*^195^^,^^325^.

Other polymeric particles are also successfully used instead of PLGA NPs. For example, dental resin composites with quaternary ammonium polyethylenimine (QPEI) NPs demonstrated antimicrobial activity against intraoral biofilm^326^, while poly(L-lactic acid) (PLLA) NPs modified by Ag particles was capable of inhibiting S. epidermidis biofilm formation^327^. Levofloxacin encapsulated into poly(caprolactone) nanoparticles (PCL NPs) was used as a component in treatment of lung infections^328^.

#### Hybrid nanoparticles

Recent progress in the field of hybrid NPs has provided new opportunities for the development of a wide range of antibacterial functional materials. For example, hybrid nanosystems such as AgNPs dispersed inside a functionalised novolac resin matrix, combining properties of both components, offer antibacterial activity towards Gram-positive and Gram-negative bacteria^329^. Lipid-coated hybrid nanoparticles (LCHNPs) are the next-generation core–shell structured nanodelivery systems, in which an inorganic or organic core, loaded with antimicrobials, is coated with lipid layers. This core–shell structure with diverse coatings may improve the capacity for loading of therapeutics, and also has the potential to improve therapeutic delivery, especially for targeting biofilm-associated and intracellular infections^330^. So, to improve the efficiency of antibiotic delivery and targeting, Yang et al. reported a unique gentamicin-loaded mesoporous silica NPs coated with bacteria toxin-responsive lipid bilayers that is decorated with the bacteria-targeting peptide, UBI^331^.

The AgNPε-polylysine nanocomposite demonstrated antibacterial effects against *P. aeruginosa* and *S. aureus* species^332^. Also hybrid NPs, formed from a PLGA nanoparticulate core with a lipid shell composed of DOTAP and DSPE-PEG5K, demonstrated biofilm-penetrating properties. Such NPs with dimensions of 100–130 nm showed a superior binding affinity to several bacterial species, including both Gram-positive and -negative pathogens. These hybrid NPs were capable often capsulating various hydrophobic antibiotics with relatively high loading efficiencies, and they gave rise to a significantly increased biofilm inhibition^332^.

Composite devices in which antibiotic-loaded silica NPs were embedded within collagen hydrogels have been described. These resulted in a delivery system that was capable of preventing bacterial infections due to a sustained delivery of antibiotic (gentamicin or rifampicin), while the outer collagen layer promoted the healing of chronic wounds^333^.

Among various nanocarriers, vesicles such as liposomes and polymersomes are considered to be promising alternatives for delivery of hydrophilic and lipophilic drugs. They have different classifications according to their composition, among which hybrid vesicles, unlike liposomes, are composed of both lipids and polymers. These vesicular systems stand out since they combine the advantages of both components, overcoming the limitations of traditional delivery systems arising from low stability and premature release of the encapsulated active substance^334^.

## Conclusion and prospects

Even though there are various strategies utilised by pharmaceutical companies and research groups worldwide to develop new antibacterial drugs, we have highlighted in our opinion one of the most novel and promising ones.

Since the discovery of penicillin natural products still remain one of very important sources for discovery of novel antibacterial agents being natural products or synthetic derivatives. Therefore, taking into account that pull of natural compounds is far from being fully investigated, natural products remain and most probable will remain of one of the sources of inspiration for the development of new antibacterial drugs in next decades.

As one of important aspects of the discovery of novel antibacterial agents is the identification of new drug targets. During last decade great attention was dedicated to bacterial metalloenzymes and carbonic anhydrases in particular. Even though these enzymes are ubiquitous in nature’s kingdom, bacterial carbonic anhydrases usually belong to different family compared to human ones. This in turn allows the development of novel inhibitors which targets bacterial metalloenzymes and will not interfere with human carbonic anhydrases. Additionally, carbonic anhydrases along different bacteria also differ, and that allows selective targeting of pathogenic bacteria and allows not killing friendly bacteria, for instance, in microbiota. Considering these facts bacterial carbonic anhydrases will be in focus for the development of novel antibacterial agents for the next decades.

Biopharmaceuticals and antisense peptide nucleic acids as novel antibacterials have gained attention in recent years. These types of compounds reduce the viability of pathogenic bacteria by selective binding to bacterial RNA, thus allowing targeting specific bacteria only. Even though there is still a lot of research to be performed, the development of this type of biopharmaceuticals is very intriguing in will take the attention of many scientists in nearest future.

Biofilm formation often is involved in bacterial infectious diseases. Biofilm formation is based on the bacterial communication system or quorum sensing. An interruption or inhibition of this communication will not only lead to treatment of disease and reducing of pathogenic bacteria, but also to the reduction of formation of bacterial resistance. This bacterial control system, where pathogenic bacteria are not directly targeted, but instead its communication is interrupted is one of the rising and probably also one of the most important strategies for bacterial control.

And last but not least antibiotic drug delivery nanosystems play a great role in the application of know antibiotics as well as novel molecules. Such nanosystems not only solve problems with drug delivery to and in pathogenic bacteria thus allowing more efficient treatment of bacterial infectious diseases which was not possible in the past, but also may solve the problem with drug availability in less developed regions of our planet, where instead of injection drug nanofurmalates might be used for slow and regular drug release.

Taking all together this review covers in our opinion one of the most aspects of the discovery of novel antibacterials.
